# Impacts of orthophosphate–polyphosphate blends on the dissolution and transformation of lead (II) carbonate

**DOI:** 10.1038/s41598-022-22683-2

**Published:** 2022-10-25

**Authors:** Javier A. Locsin, Benjamin F. Trueman, Evelyne Doré, Aaron Bleasdale-Pollowy, Graham A. Gagnon

**Affiliations:** 1 Department of Civil and Resource Engineering, Centre for Water Resources Studies, Dalhousie University, 1360 Barrington St., Halifax, NS B3H 4R2 Canada; 2 Halifax Water, 450 Cowie Hill Road, Halifax, NS B3P 2V3 Canada

**Keywords:** Environmental impact, Engineering

## Abstract

Orthophosphate–polyphosphate blends are commonly used to control lead release into drinking water, but little is known about how they interact with lead corrosion scale. Conventional corrosion control practice assumes that orthophosphate controls lead release by forming insoluble Pb-phosphate minerals, but this does not always occur, and under certain conditions, phosphate blends may increase lead release. Here, we used continuously-stirred tank reactors to compare orthophosphate–polyphosphate blends with orthophosphate on the basis of lead (II) carbonate dissolution and transformation at environmentally relevant phosphate concentrations. Three model polyphosphates—tripoly-, trimeta- and hexametaphosphate—were used. Hexametaphosphate was the strongest complexing agent (1.60–2.10 mol_Pb_/mol_Polyphosphate_), followed by tripolyphosphate and trimetaphosphate (1.00 and 0.07 mol_Pb_/mol_Polyphosphate_, respectively. At equivalent orthophosphate and polyphosphate concentrations (as P), orthophosphate-trimetaphosphate had minimal impact on lead release, while orthophosphate-tripolyphosphate increased dissolved lead. Orthophosphate-hexametaphosphate also increased dissolved lead, but only over a 24-h stagnation. Both orthophosphate-tripolyphosphate and orthophosphate-hexametaphosphate increased colloidal lead after 24-h. Increasing the concentrations of hexameta- and tripoly-phosphate increased dissolved lead release, while all three polyphosphates inhibited the formation of hydroxypyromorphite and reduced the phosphorus content of the resulting lead solids. We attributed the impacts of orthophosphate–polyphosphates to a combination of complexation, adsorption, colloidal dispersion, polyphosphate hydrolysis, and lead mineral precipitation.

## Introduction

While many drinking water utilities add phosphate chemicals for corrosion control or sequestration, revisions to the Lead and Copper Rule (LCR)^1^ will increase the number of utilities evaluating, changing, and implementing phosphate treatment. Utilities may need to provide both adequate sequestration and optimized corrosion control using blends of orthophosphate and polyphosphate. The use of polyphosphate and orthophosphate–polyphosphate blends for corrosion control in drinking water distribution systems is controversial. While polyphosphate is effective for controlling aesthetic water quality issues (i.e. discoloration, calcium scaling)^2,3^, it increases lead release through aqueous complexation or colloidal dispersion^4–6^, and lead-polyphosphate complexes have been observed in tap water^7^.

While the impact of orthophosphate (OrthoP) on lead corrosion is well studied^6,8,9^, the mechanisms by which orthophosphate–polyphosphate blends act to limit lead (Pb) release are unclear. One major problem is that the formulation of orthophosphate–polyphosphate blends is generally proprietary, with orthophosphate concentrations ranging between 5 and 70%^10^. Moreover, the precise concentrations of specific polyphosphate species are not always known, even to the manufacturer^11^, and hydrolysis of polyphosphate in the distribution system can result in a variable mixture of OrthoP and smaller polyphosphates, further confounding the effects of each phosphate species.

Individual polyphosphates may differ widely in their effects on water quality: structural differences strongly determine polyphosphate interactions with metal ions. Linear polyphosphates (e.g. tripolyphosphate) are more effective sequestrants than cyclophosphates (e.g. trimetaphosphate, hexametaphosphate)^12,13^. McGaughey^14^ showed that trimetaphosphate was least effective at preventing calcium precipitation compared to tripolyphosphate or hexametaphosphate and it inhibited the dissolution of hydroxyapatite, whereas the other two polyphosphates solubilized the mineral. This may be due to steric constraints characteristic of cyclophosphates that can be mitigated by increased chain length^15^. Fundamental polyphosphate chemistry predicts that, at equivalent chain lengths, cyclophosphates will have a smaller effect on lead release than linear polyphosphates and that lead release will increase with polyphosphate chain length.

In addition, theoretical lead solubility predictions with blended phosphates are difficult due to the lack of solubility and formation constants as well as a limited understanding of their effects on lead corrosion scale formation. While the orthoP component is expected to form an insoluble lead-phosphate, this is not always observed in lead pipe scale^11,16^. Instead, field studies have documented complex amorphous layers of phosphorus and co-precipitated metals (e.g. Al, Ca, Fe) forming in orthophosphate–polyphosphate treated systems^11,16^. While corrosion scale plays an important role in lead release, mineral formation due to blended phosphate has not been systematically studied. A limited understanding of orthophosphate–polyphosphate–lead interactions leaves utilities that use these additives vulnerable to lead contamination at consumers’ taps.

To address these knowledge gaps, we studied lead carbonate dissolution in the presence of orthophosphate and polyphosphate. The goals of this work were to:

1. Compare lead release, quantified as the net conversion from suspended lead (II) carbonate to dissolved (< 0.2 µm) and small lead colloids (0.2–0.45 µm), in the presence of the three representative polyphosphates: tripolyphosphate (TripolyP), trimetaphosphate (TrimetaP), and hexametaphosphate (HexametaP). Each was tested alone and blended with orthophosphate, using a continuous-flow stirred-tank reactor (CSTR).
2. Evaluate mineral formation in the presence of OrthoP in combination with TripolyP, TrimetaP, or HexametaP.

## Materials and methods

### Preparation of solutions

We used ultrapure water (18.2 MΩ cm, TOC < 2 µg L^−1^) to prepare all solutions in this study. Solution composition was chosen to reflect drinking water conditions with low inorganic carbon content. All chemicals were reagent grade or better. Sodium hexametaphosphate ((NaPO_3_)_6_) (Alfa Aesar, Haverhill, MA), sodium trimetaphosphate ((NaPO_3_)_3_) (Alfa Aesar, Haverhill, MA), and sodium tripolyphosphate (Na_5_P_3_O_10_) (Alfa Aesar, Haverhill, MA) were used to represent different polyphosphate structures. Polyphosphate stock solutions were obtained by dissolving TripolyP, TrimetaP, or HexametaP in 100 mL ultrapure water, before each experiment. All polyphosphate solutions were prepared and used the same day. OrthoP was added as ACS grade phosphoric acid (Fisher Chemical, Fairlawn, NJ). The dissolved inorganic carbon (DIC) concentration of 5 mg C L^−1^ was achieved by dissolving sodium bicarbonate powder (Fisher Chemical, Fairlawn, NJ) in 20 L of ultrapure water. The pH was adjusted by the addition of 1 N trace metal grade nitric acid (Fisher Chemical, Fairlawn, NJ) or freshly prepared 2 N sodium hydroxide (Fisher, Fairlawn, NJ).

### Flow-through reactor Pb dissolution experiments

**Figure 1:**
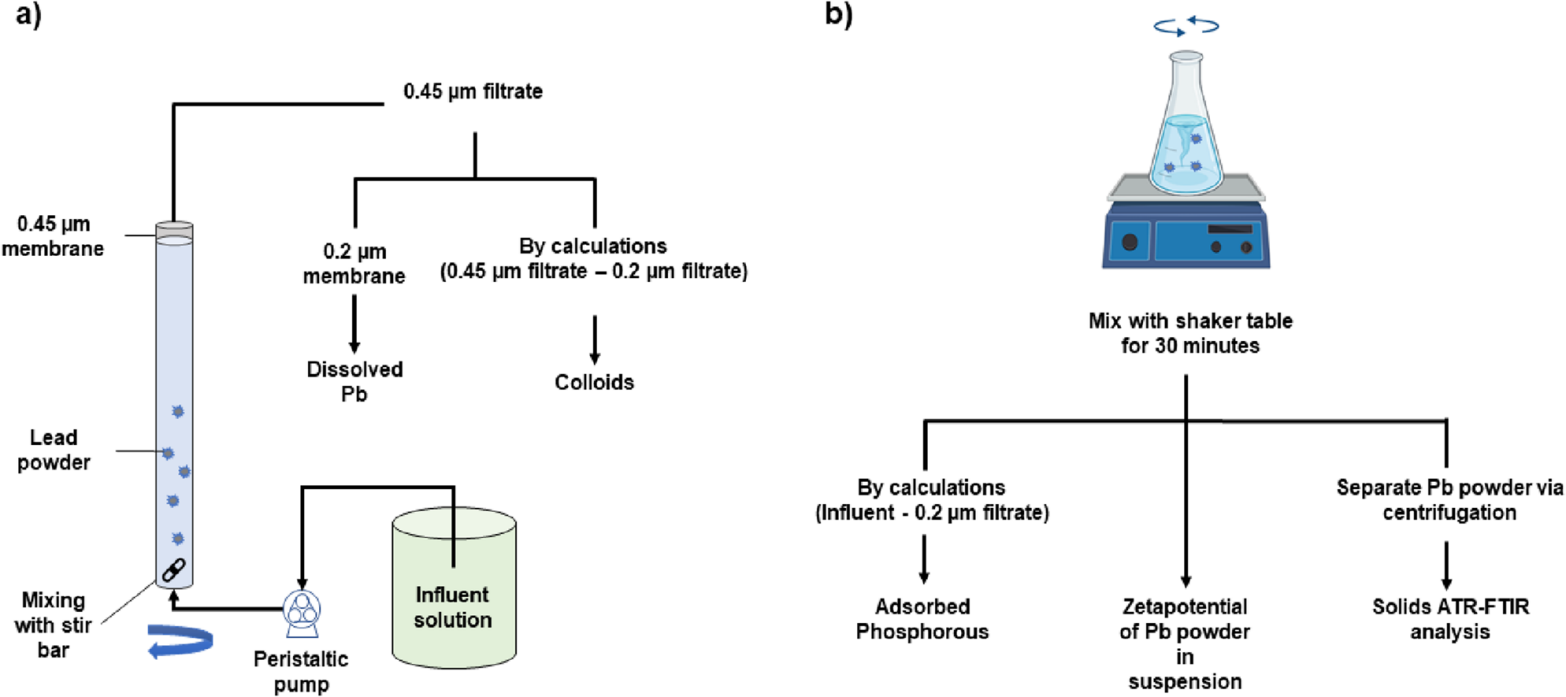
A schematic overview of the (**a**) CSTR assembly used in dissolution experiments and (**b**) batch reactor used in the adsorption experiments. Effluent from the CSTR was separated into 0.45 µm filtrate, colloids (0.2–0.45 µm), and dissolved (< 0.2 µm) lead.

We used continuously stirred tank reactors (CSTR) made with glass columns (Kimble, Rockwood, TN, 144 mL) to evaluate the impacts of phosphate composition, calcium, and pH on lead solubility and mineral formation (Fig. 1). Before each experiment, all columns, tubing (Masterflex LS-14, internal diameter = 1.6 mm), stir bars, and glass carboys were immersed in dilute HNO_3_ (~ 1.6 M) for 24 h and rinsed at least four times with ultrapure water. Experiments were carried out in duplicate at room temperature (21 ± 2 °C). The CSTRs were fed using peristaltic pumps (Cole Palmer, Montreal, QC) and capped with 0.45 µm cellulose nitrate membranes (Whatman, Maidstone, UK) to prevent the loss of lead solids from the reactor (Fig. 1). The influent flow rate was maintained at 4.9 mL min^−1^ using peristaltic pumps, providing a hydraulic retention time (HRT) of 30 min. This was chosen to reflect current Canadian and European lead sampling guidelines^17,18^. To ensure saturation, 1 g L^−1^ of lead (II) carbonate powder (Alfa Aesar, Haverhill, MA), composed of a mixture of cerussite and PbCO_3_·PbO^6^, used as a surrogate of lead corrosion scale, was suspended in the reactors.

Previous studies have noted the high variability in lead release due to labile lead phases in the first 48 HRTs^6,8^. Based on previous work^6^, reactors were considered stable and sampled after 58 HRTs. At the end of the flow-through experiment, the influent was shut off, reactors were sealed with plastic caps and left to mix for 24 h (24HS) to evaluate the effect of long stagnation times on lead release.

Lead release was quantified as mass released per unit surface area under (a) steady-state at a 30-min HRT, or (b) at the end of the 24-h stagnation. Here, steady-state was defined as less than 30% variation (standard deviation/mean) in lead concentrations over at least four consecutive effluent samples, spanning at least eight HRTs. Once the reactor effluent had stabilized, mass release per unit surface area was calculated according to Eq. (1)

1 $${r}_{exp}(\mathrm{\mu g} {m}^{-2})=\frac{Css}{SA*Pbsolid}$$

where ${r}_{exp}$ is the mass release per unit surface area (SA) (µg m^−2^), ${C}_{ss}$ is the effluent lead concentration (µg L^−1^, SA is the surface area of lead powder determined to be 0.78 m^2^ g^−1^, and $P{b}_{solid}$ is the mass of lead powder in g L^−1^.

The effluent was further filtered through a 0.2 µm polycarbonate membrane (Whatman, Maidstone, UK) to get dissolved lead using a syringe apparatus. The 0.2 µm membranes were decontaminated with dilute nitric acid (~ 0.16 M), washed with 10 mL ultrapure water, then pre-conditioned with 10 mL of sample to minimize losses to adsorption^6^.

Since at least 8.6 L of reactor effluent passed through the 0.45 µm cellulose nitrate membrane before sample collection, adsorption losses to that membrane were expected to be minimal^6^.

### Experiment design

We first evaluated the performance of four phosphates at 1000 µg P L^−1^—OrthoP, TripolyP, TrimetaP, and HexametaP—on lead release in the CSTR at pH 7.5 ± 0.2. We then investigated the effect on lead release of two factors—orthophosphate–polyphosphate composition (OrthoP-TripolyP, OrthoP-TrimetaP, or OrthoP-HexametaP), and OrthoP (300 µg P L^−1^) to polyphosphate (300 (1:1) or 700 (1:2) µg P L^−1^) concentrations—against OrthoP at 300 µg P L^−1^ at pH 7.5 ± 0.2.

### Short term phosphate adsorption by lead carbonate solids—batch experiment

To support the CSTR experiment, the effect of OrthoP or polyphosphate on phosphorus adsorption, quantified as the difference between dissolved (< 0.2 μm) phosphorus at the beginning and end of the 30 min reaction, was concurrently measured with lead dissolution in a batch reactor. In a 250 mL Erlenmeyer flask, a 1 g L^−1^ dispersion of lead (II) carbonate was prepared in a 5 mg L^−1^ DIC solution. The roles of phosphate composition were explored by adding 1000 µg P L^−1^ of either OrthoP, TripolyP, TrimetaP, or HexametaP at two pH (7 or 9). These pH would represent the upper and lower bound of pH at which phosphate inhibitors are used in drinking water^4,19^. The effects of different combinations of OrthoP (1000 µg P L^−1^) with each of the polyphosphates (500–2000 µg P L^−1^) were investigated at pH 7. The suspensions were shaken mechanically for 30 min, at 150 rpm and room temperature (21 ± 2 °C). Thirty minutes was chosen to both mirror stagnation in the CSTR experiment and minimize polyphosphate hydrolysis to OrthoP^20^. After 30 min, an aliquot was taken from the vessel and filtered through a 0.2 µm polycarbonate membrane. The filtrates were analyzed for dissolved phosphorus and lead via inductively coupled plasma mass spectrometry (ICP-MS).

### Effect of phosphates on lead carbonate interparticle forces—batch experiment

The electrophoretic mobility of lead carbonate particles in solution (2 g L^−1^) was measured as a function of pH (5–9.5) and phosphate type-OrthoP, TripolyP, TrimetaP, and HexametaP- at 1000 µg P L^−1^. The solution pH was controlled within 0.2 pH units. The solution was initially set to approximately pH 9.5, after which the pH was gradually decreased to 5 via the addition of 1 N HNO_3_. Independent reactors, run in duplicate, were used.

### Analytical methods

#### Element quantification

Metals were quantified by ICP-MS (X Series II, Thermo Fisher Scientific, Waltham, MA) using Standard Methods 3125 and 3030 (American Public Health Association, American Water Works Association, Water Environment Federation, 2012). Reporting limits for lead and phosphorus were 0.4 and 4.9 µg L^−1^, respectively. Filtered effluent was acidified to pH < 2 with concentrated trace metal grade HNO_3_ and held for a minimum of 24 h, at room temperature, before analysis^21^. Dissolved phosphate (PO_4_) concentrations were measured using EPA 300.1^22^ via ion chromatography (Dionex Aquion IC with AS22 column, Thermo Fisher Scientific, MA) with a reporting limit of 10 µg L^−1^ phosphate.

Dissolved metals were defined as lead passing through a 0.2 µm membrane. The colloid (0.2–0.45 µm) fraction was calculated by subtracting dissolved from the 0.45 µm filtrate (Fig. 1). Particles smaller than 0.2 µm may be present in the dissolved fraction.

#### Structural characterization of Pb particles

Scanning electron (SEM) (Hitachi S-4700, Tokyo, Japan) microscopy with energy dispersive X-ray spectroscopy (EDS) was used to observe the morphology and analyze elemental composition of powder surfaces. X-ray diffraction (XRD) (Rigaku Ultima IV X-ray Diffractometer, The Woodlands, TX, copper K$\alpha$ source) was used to identify crystalline phases. Diffraction patterns were analyzed using Match! 3, version 3.9.0.158^23^ software and Crystallographic Open Database Rev.218120 database. The surface area of the lead (II) carbonate powder was determined via Brunauer–Emmett–Teller analysis (BET-N2) (Nova 4200E, Quantachome, Boynton Beach, FL).

#### Infrared spectroscopy

A single-beam Fourier transform infrared spectroscopy in attenuated total reflectance mode (ATR-FTIR) (Bruker alpha-P, Billerica, MA) was used. Each infrared spectrum (IR) was recorded with the blank cell as the background. Fifty scans at a wavenumber range between 400 and 4000 cm^−1^ were measured to obtain each spectrum, with a resolution of 4 cm^−1^. IR spectra of dissolved phosphate species were measured by using a phosphate solution, composed of either OrthoP or polyphosphate in ultrapure water at pH corresponding to the experimental conditions. The pure water IR spectrum was subtracted to produce the spectra of dissolved phosphate species.

At the end of the flow-through experiment, the resulting lead powder was dried in a desiccator for 1 week. Samples were spread evenly across the surface of the ATR crystal using a plastic spatula and their IR spectra were recorded. At the end of the batch adsorption experiment, the spectra of adsorbed phosphate species on the Pb surface were measured using the phosphate solution in the Pb suspension as the sample and the same suspension, without phosphate, as the reference.

#### Electrophoresis

Zeta potentials were measured using a Zetasizer Nano ZS (Malvern Panalytical, Malvern, UK). Aliquots of lead (II) carbonate suspension were extracted and measured after a 5 min reaction period at the corresponding pH.

### Data analysis

We used R, along with contributed packages, for data analysis and presentation^24,25^. All experiments were duplicated, with lead concentrations expressed as medians with ranges provided in parenthesis. We used a Hodges-Lehmann estimate to quantify the difference between dosed and recovered phosphorus in the batch experiments^26^.

## Results and discussion

### Impacts of orthophosphate or polyphosphate on lead release in CSTR experiments

**Figure 2:**
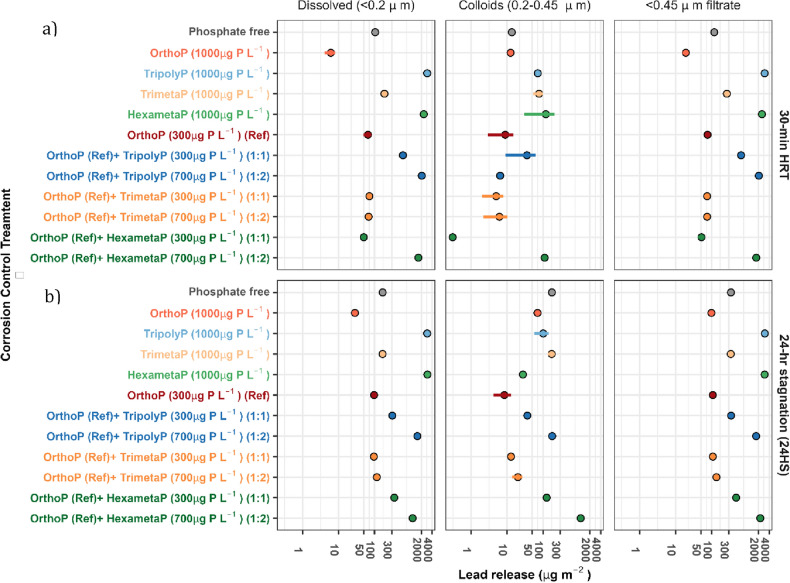
Median mass lead release per unit surface area (log-scale) during a (**a**) 30 min and (**b**) 24-h reaction at pH 7.5 and DIC 5 mg C L^−1^ from the CSTR. We compared lead release from a phosphate free condition, orthophosphate (1000 µg P L^−1^) and three polyphosphates (1000 µg P L^−1^). We also compared blends of orthophosphate (300 µg P L^−1^) and polyphosphates (300 or 700 µg P L^−1^) against orthophosphate (300 µg P L^−1^). Each set represents at least two reactor runs, and error bars span the median absolute deviation of measurements. Tabulated data can be found in Table S1.

As expected, OrthoP decreased median lead concentrations compared to the phosphate-free condition. With an HRT of 30 min, OrthoP treatment decreased lead in 0.2 µm filtrate (dissolved) by an estimated 98.1 (range = 91.6–99.3) µg Pb_0.2 µm_ m^−2^, lead in 0.45 µm filtrate by 99.1 (95.3–104.9) µg Pb_0.45 µm_ m^−2^, while colloidal lead—the difference between 0.45 and 0.2 µm filtrate—did not vary significantly (Fig. 2a). With a 24-h stagnation period (24HS), OrthoP treatment decreased lead in 0.2 µm filtrate by 139.9 (130.8–149.0) µg Pb_0.2 µm_ m^−2^, lead in 0.45 µm filtrate by 245.6 (229.2–262.0) µg Pb_0.45_ _µm_ m^−2^, and colloidal lead by 105.7 (98.3–112.0) µg Pb_0.2–0.45 µm_ m^−2^ (Fig. 2b).

TripolyP, the linear polyphosphate, yielded the highest lead concentrations among the polyphosphates, and the dissolved fraction was dominant (> 97%) (Fig. 2a,b). Compared to the phosphate-free condition, median lead release increased by 2847 (2837–2849) µg Pb_0.45 µm_ m^−2^ and 2664 (2640–2667) µg Pb_0.45 µm_ m^−2^ at a 30-min HRT and 24HS, respectively. TripolyP had a potential complexation capacity—calculated as the molar ratio of median dissolved lead and median residual polyphosphate—of 1.00 ± 0.01 mol_Pb_/mol_TripolyP_ (at both a 30-min HRT and 24HS). Moreover, TripolyP achieved apparent equilibrium within 30 min. That is, Pb_0.45 µm_ concentrations between a 30-min HRT and 24HS did not vary by more than 38.5 (2.60–84.6) µg Pb_0.45 µm_ m^−2^_._

TrimetaP, a cyclophosphate with equivalent chain length, had the least impact on lead concentrations among the polyphosphates, with a potential complexation capacity of 0.07 ± 0.01 mol_Pb_/mol_TrimetaP_ (at both retention times). Lead release due to TrimetaP was similar to that representing the phosphate-free condition at 24HS. At a 30-min HRT, lead release was higher by 145.7 (141.4–149.7) µg Pb_0.45 µm_ m^−2^.

When HexametaP, the larger cyclophosphate, was added instead, lead complexation capacity was greater than TrimetaP: 1.60–2.10 ± 0.01 mol_Pb_/mol_HexametaP_ (30-min HRT and 24HS). This is consistent with previous work describing lead release from pipes in the presence of HexametaP^4^. Lead release was mostly dissolved (> 89%) and exceeded that representing the phosphate-free control by an estimated 2352 (2248–2435) µg Pb_0.45 µm_ m^−2^ and 2626 (2559–2693) µg Pb_0.45 µm_ m^−2^ at a 30-min HRT and 24HS, respectively.

OrthoP and cyclophosphates dispersed colloidal lead at a 30-min HRT (Fig. 2a). OrthoP treatment resulted in a higher proportion of small colloids compared to polyphosphates (65% vs 2.4% vs 29.2% vs 4.8% of Pb_0.45_ μm for OrthoP, TriployP, TrimetaP, and HexametaP, respectively). While the proportion of small colloids increased with stagnation time (24HS) for OrthoP (71.1% of Pb_0.45 µm_) and TrimetaP (50.8% of Pb_0.45 µm_) treatment, it was similar with TripolyP and decreased with HexametaP (0.9% of Pb_0.45 µm_). Lead colloids dispersed by HexametaP at the 30-min HRT appeared to dissolve over the extended stagnation (24HS), based on the large decrease in the colloidal fraction with increasing reaction time.

Excess OrthoP is known to promote Pb colloid formation, especially above a P:Pb molar ratio of 1^9^. At 1000 µg P L^−1^ OrthoP, the P:Pb molar ratio of 302:1 (32.3 µM P: 107 nM Pb) would encourage lead-phosphate precipitation. Polyphosphates also promote colloid formation: TrimetaP can impart a negative surface charge (See “Impacts on electrical double layer and adsorption of orthophosphate or polyphosphate”) and has been observed to promote apatite formation even at relatively low pH^27^. HexametaP can also stabilize colloids^6^.

### Combining orthophosphate and polyphosphate

Median lead concentrations in the < 0.45 µm filtrate from reactors dosed with OrthoP alone were 77.1 (61.9–96.4) and 108.0 (101.3–113.1) µg Pb_0.45 µm_ m^−2^ at a 30-min HRT and 24HS, respectively. Dissolved lead concentrations were 65.1 (34.5–83) and 97.1 (94.7–101.1) µg Pb_0.2 µm_ m^−2^_,_ while colloidal lead concentrations were 8.90 (6.70–14.5) and 8.40 (4.20–12.2) µg Pb_0.2–0.45 µm_ m^−2^ (Fig. 2a,b).

Blending either TripolyP or HexametaP with OrthoP increased lead release relative to the OrthoP-only control. Adding TripolyP increased dissolved lead at both doses and stagnation times (1:1 orthophosphate:polyphosphate ratio: 554.0 (540.3–572.8) and 213.5 (191.4–235.5) µg Pb_0.2 µm_ m^−2^ at 30-min HRT and 24HS, respectively; 1:2 ratio: 1981 (1936–2005) and 1453 (1445–1459) µg Pb_0.2 µm_ m^−2^) (Fig. 2a,b). Adding HexametaP increased dissolved lead at 24HS (256.3 (243.1–269.7) vs 1046 (958.7–1134) µg Pb_0.2 µm_ m^−2^ at 1:1 and 1:2 ratios, respectively).

Both OrthoP-TripolyP and OrthoP-HexametaP increased small colloidal lead concentrations at 24HS. Compared to a 30-min HRT, 700 µg L^−1^ of TripolyP (1:2 ratio) accompanied a decrease in dissolved lead of 502.4 (471.8–510.0) µg Pb_0.2 µm_ m^−2^ and an increase of 173.7 (144.9–190.8) µg Pb_0.2–0.45 µm_ m^−2^ in small colloidal lead. After 24HS, OrthoP-HexametaP treatment increased small colloid concentrations by 124.0 (119.4–131.7) and 980.0 (831.3–1102) µg Pb_0.2–0.45 µm_ m^−2^ at the 1:1 and 1:2 ratios, respectively.

**Figure 3:**
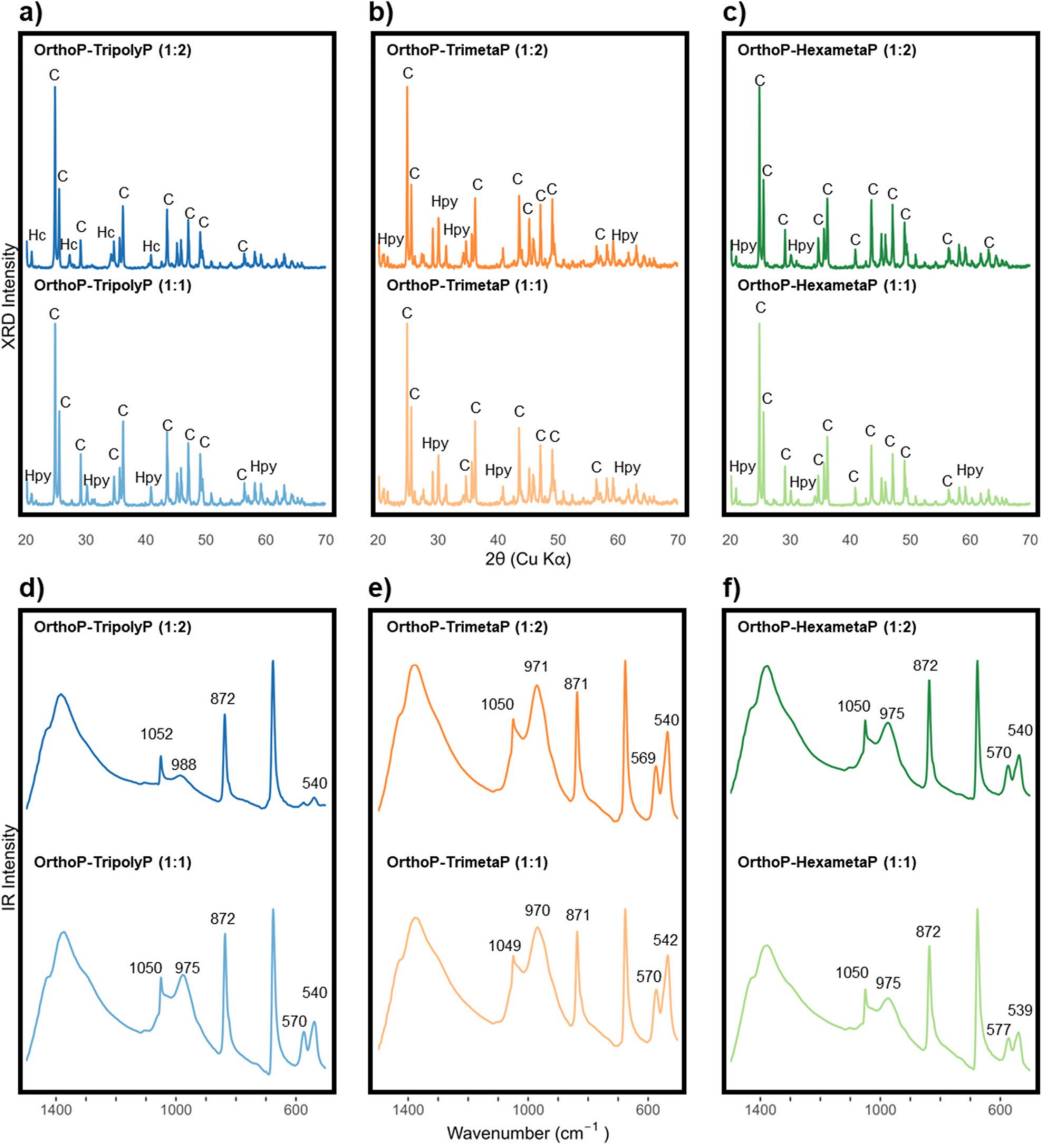
(**a**–**c**) XRD and (**d**–**f**) FTIR spectra of lead particles from CSTR experiments after reactions with orthophosphate-tripolyphosphate, orthophosphate-trimetaphosphate, and orthophosphate-hexametaphosphate at pH 7.5 and DIC 5 mg C L^−1^. Prominent mineral or IR peaks are identified above. XRD lead mineral peaks are identified as follows: C—cerussite, Hc—hydrocerussite, and Hpy—hydroxypyromorphite.

**Figure 4:**
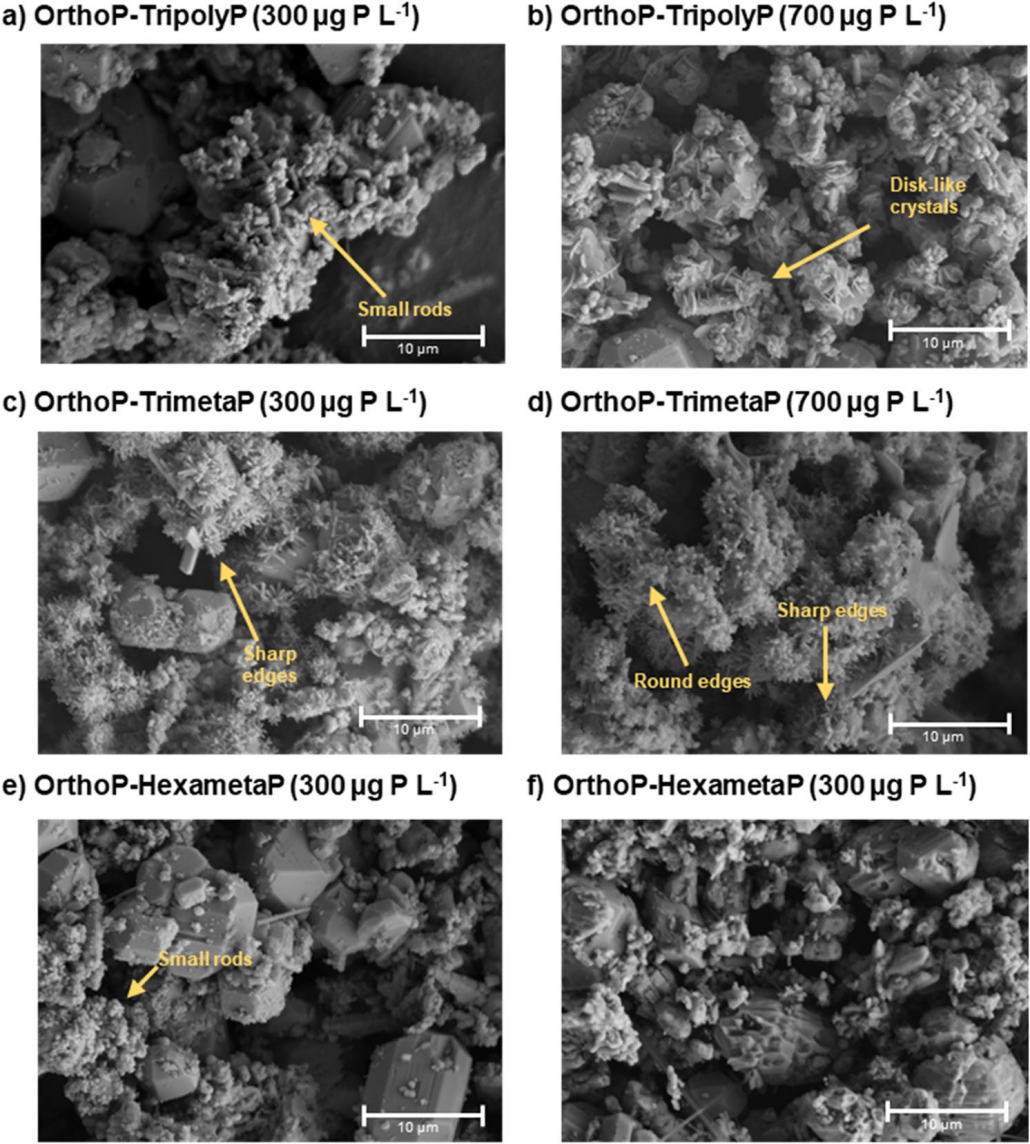
SEM images of lead particles from CSTR experiments after reactions with (**a**, **b**) orthophosphate-tripolyphosphate, (**c**, **d**) orthophosphate-trimetaphosphate, and (**e**, **f**) orthophosphate-hexametaphosphate at pH 7.5 and DIC 5 mg C L^−1^.

Blending TripolyP or HexametaP with OrthoP inhibited the formation of hydroxypyromorphite. At equivalent OrthoP:polyphosphate concentrations (1:1), XRD peaks (Fig. 3a–c), IR peaks (~ 540 and 570 cm^−1^) (Fig. 3d–f), and rod-like crystals^28^ (Fig. 4a,e) characteristic of hydroxypyromorphite were observed. At the higher TriployP concentration (1:2), hydroxypyromorphite was no longer observed. Instead, disk-like crystals^29^ (Fig. 4b) and XRD peaks characteristic of hydrocerussite were seen (Fig. 3a, Fig. S1). Furthermore, the proportion of adsorbed phosphorus measured via energy dispersive X-ray spectroscopy (EDS) decreased from 41.69 ± 3.74% to 1.55 ± 1.41% (weight %) as TripolyP was increased from 300 to 700 µg P L^−1^ (OrthoP-TripolyP at 1:1 and 1:2 ratios, respectively). Similarly, increasing the HexametaP concentration by the same mass (1:1–1:2) resulted in the disappearance of diffraction peaks characteristic of hydroxypyromorphite (2*θ* = 22, 26.9, 30.5, 32.4, and 58.8°) (Fig. 3b, Fig. S3) and small rod-like crystals (Fig. 4f). This result was further supported by a decrease in the proportion of phosphorus on the lead solid from 13.79 ± 3.46 (1:1) to 0.24 ± 0.11 wt% (1:2).

OrthoP-TrimetaP had minimal impact on lead release at both stagnation times (Fig. 2a,b). At a 1:1 ratio, median lead release in the presence of OrthoP-TrimetaP differed from OrthoP by just 2.0 and 9.3 µg Pb_0.45 µm_ m^−2^ whereas lead release at a 1:2 ratio was greater by just 1.9 and 28.0 µg Pb_0.45 µm_ m^−2^ at a 30-min HRT and 24HS, respectively. A 1:1 blend of OrthoP and TrimetaP decreased lead release at 24HS by 239.4 (209.8–269.1) and 372.7 (347.9–395.4) µg Pb_0.45 µm_ m^−2^ compared to the equivalent blends with TriployP or HexametaP, respectively. Also, the inhibition of hydroxypyromorphite formation with TrimetaP was not as extreme as with TripolyP or HexametaP. While XRD (Fig. 3a–c) and IR peaks (539–546 and 570–577 cm^−1^) (Fig. 3d–f) characteristic of hydroxpyromorphite were observed across all OrthoP-polyphosphates at the 1:1 ratio, their intensities were lower with HexametaP or TripolyP compared to TrimetaP. Hydroxypyromorphite was clearly visible (Figs. 3b, 4c) with OrthoP-TrimetaP treatment (1:1 ratio) but increasing the TrimetaP concentration (1:2) appeared to inhibit its formation (Fig. 4d). Hydroxypyromorphite crystals at the 1:1 ratio presented rod-like particles with sharp, defined edges (Fig. 4c), whereas crystals with the 1:2 ratio were still rod-like but presented round edges (Fig. 4d). Moreover, phosphorus in the lead solid was reduced from 25.8 ± 7.3% to 6.4 ± 2.4% (weight %) when TrimetaP was increased from 300 (1:1) to 700 (1:2) µg P L^−1^.

### Exploration of interaction mechanisms for Pb and phosphates

#### Impacts on electrical double layer and adsorption of orthophosphate or polyphosphate

In a batch experiment, we examined the surface charge and adsorption behavior of phosphorus onto lead (II) carbonate at pH 7 (0 ± 0.14 mV, mean ± standard deviation) and 9 (− 25.2 ± 0.3 mV) to allow for both neutral and negatively charged surfaces, respectively. Of particular interest was the effect of chain length and structure on phosphate adsorbed on the lead surface.

**Figure 5:**
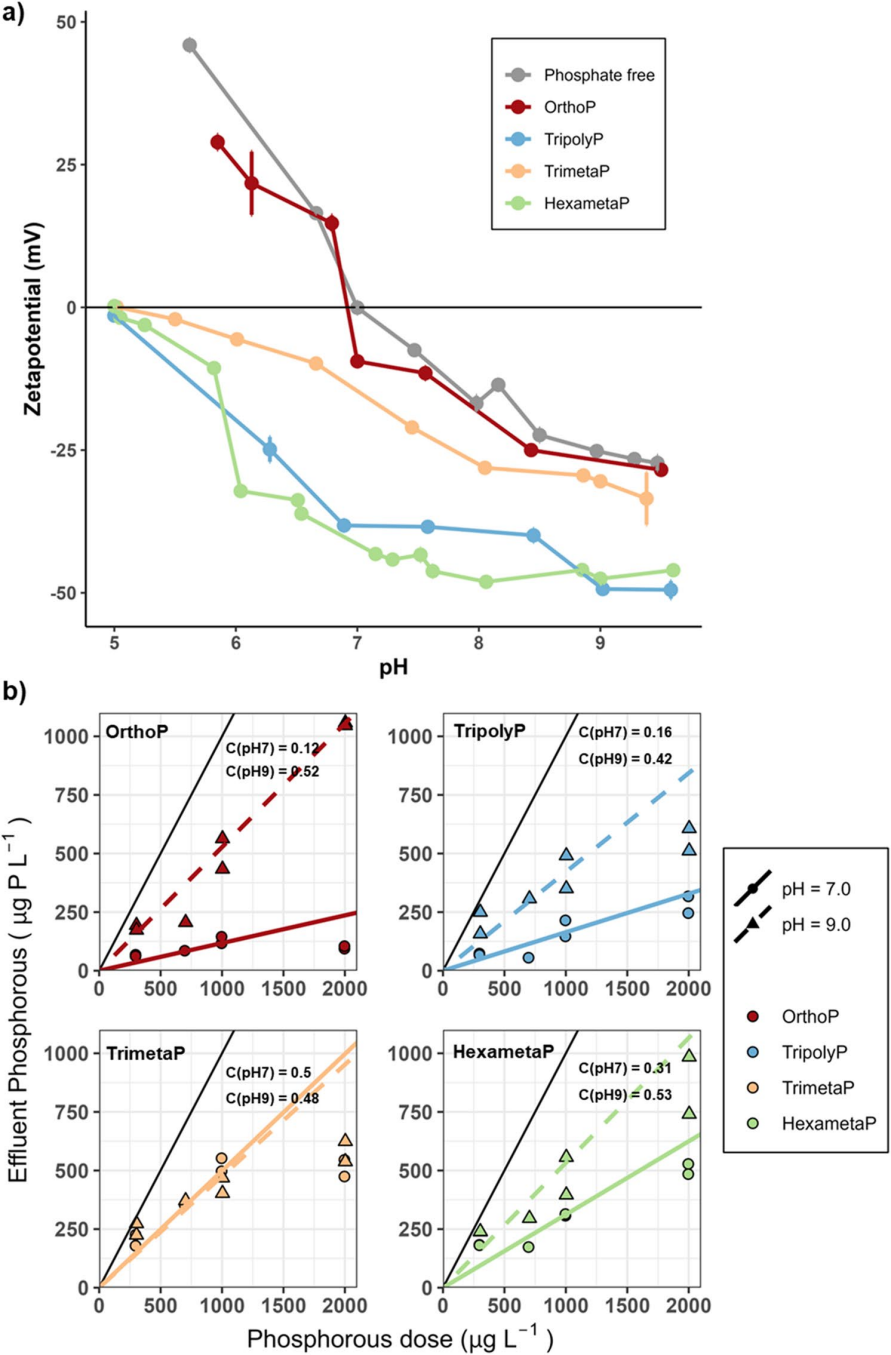
(**a**) Zetapotential (pH = 5.0–9.5, P = 0 or 1000 µg P L^−1^), (**b**) phosphorus adsorption (pH = 7.0 or 9.0, P = 0–2000 µg P L^−1^) by lead carbonate as a function of pH and phosphate type (OrthoP, TripolyP, TrimetaP, HexametaP) at DIC 5 mg C L^−1^ from the batch experiment. Each set represents at least two reactor runs, and error bars span the median absolute deviation of measurements. Colored lines (representing y = Cx) are labeled by their corresponding C, where C is the estimated fraction of total phosphorus remaining in solution at the end of 30 min. The solid black line represents y = x. The estimates (C) were obtained by computing an additive difference estimate between the log-transformed influent and effluent phosphorus concentrations, resulting in a multiplicative estimate on the measurement scale.

Phosphates shifted the isoelectric potential (pH_iep_) of lead carbonate. At equivalent chain lengths, TripolyP imparted greater charge reversal than TrimetaP (Fig. 5a). Although only present at half the molar concentration, HexametaP imparted more negative charge to the lead surface than TrimetaP, suggesting that chain length is important.

Similarly, the reduction in effluent dissolved (< 0.2 µm) phosphorus (P_reduction_), measured as the ratio of effluent to influent phosphorus, presented an apparent dependence on structure and chain length (Fig. 5b). The estimates for P_reduction_ were obtained by computing an additive difference estimate between the log-transformed influent and effluent phosphorus concentrations^26^. At pH 7.0, P_reduction_ was greatest for OrthoP − 0.12, or 12% of initial P remained after reaction (95% confidence interval (CI) 0.05–0.21). At equivalent chain lengths, P_reduction_ was greater with TripolyP (0.16, 95% CI 0.11–0.23) than TrimetaP (0.50, 95% CI 0.27–0.64). Increased cyclophosphate chain length resulted in less P_reduction_: HexametaP adsorption by molar basis of polyphosphate was 2.60-fold (95% CI 1.50–4.50) lower compared to TrimetaP. The similar, albeit reduced, phosphorus adsorption observed at pH 9.0 could be attributed in part to electrostatic repulsion. At pH 7.0, lead (II) carbonate is neutrally charged whereas both lead (II) carbonate and phosphate are negatively charged at pH 9.0, likely resulting in more adsorption at pH 7.0 and repulsion at pH 9.0 (Fig. 5a). Moreover, hydroxypyromorphite is more soluble at pH 9.0. At pH 7.5 at which the CTSR experiment was conducted, phosphate species would be more deprotonated^30,31^, and the lead (II) carbonate would have a slightly more negative surface charge (− 7.5 ± 0.65 mV) than at pH 7.0. For instance, orthophosphate would comprise of 59% H_2_PO_4_^−^ and 41% HPO_4_^2−^, and 31% H_2_PO_4_^−^ and 69% HPO_4_^2−^ at pH 7.0 and 7.5, respectively. While this may result in reduced phosphorus adsorption in the CSTR, the adsorption trend seen in the short-term batch experiment can be extended to the CSTR. The apparent independence of trimetaphosphate adsorption from pH may be due to steric constraints, rather than trimetaphosphate species, reducing its ability to interact with the lead (II) carbonate surface (See “Chemical surface interactions between lead and phosphates”).

### Chemical surface interactions between lead and phosphates

To understand the interactions between phosphates and lead carbonate in the CSTR experiments, a deeper analysis of the surface properties of the lead carbonate after phosphate adsorption in a complementary batch experiment was carried out via ATR-FTIR. IR spectra of free phosphates in solution exhibit several characteristic peaks (Table 1). Following surface adsorption, IR bands experience either shifting or changes in intensity and are discussed below. The peak assignments for the ATR-FTIR spectra of adsorbed phosphates on lead carbonate are extrapolated based on the data of condensed phosphates adsorption on titania, serpentine, and metal (hydr)oxides^31–35^. A detailed description of the ATR-FTIR analysis can be found in the Supplemental material (Sects. S1 and S2).

**Table 1:** Assignment of ATR-FTIR peaks for phosphates in solution based on Guan et al.^32^, Lu et al.^33^, Michelmore et al.^31^, Socrates^34^, and Wan et al.^35^.

Phosphate type	pH	Species	Wavenumber (cm^−1^)	Assignment (free in solution)
Orthophosphate	3.7	H_2_PO_4_^−^	1157	v_as_(P–O)
			1075	v_s_(P–O)
			940	v_as_(P–OH)
			872	v_s_(P–OH)
	9.1	HPO_4_^2−^	1075	v_as_(P–O)
			990	v_s_(P–O)
			850	v_s_(P–OH)
	12.5	PO_4_^3−^	1008	v_as_(P–O)
Tripolyphosphate	6–9		1212	v_as_(P–O)
			1191–1199	v_as_(P–O in terminal HPO_3_ and H_2_PO_3_)
			1120	v_as_(P–O) in PO_3_
			1101	v_as_(P–OH in H_2_P_3_O_10_)
			1060	v_s_(P–OH H_2_P_3_O_10_)
			1029	v_s_(P–O in terminal PO_3_)
			1002	v_s_(P–OH)
			975	v_as_(P_2_O_7_^4−^)
			905	v_as_(P–O–P)
Trimetaphosphate	6–9		1260	v_as_(P–O)
			1150	v_s_(P–O)
			1086	v_s_(P–O)
			1002	v_b_(P–O)
			902	v_as_(P–O–P)
Hexametaphosphate	6–9		1260	v_as_(P–O)
			1115	v_s_(P–O)
			1090	v_as_(P–O)
			1030	v_b_(P–O)
			1008	v_b_(P–O)
			882	v_as_(P–O–P)
			868	v_s_(P–O–P) in long chain

v_as_—asymmetric stretching vibration, v_s_—symmetric stretching vibration, v_b_—bending vibration.

Lower lead release in the presence of cyclophosphates (Trimetap and HexametaP) compared to linear polyphosphate (TripolyP) can likely be explained by fundamental chemistry. First, TripolyP (5 oxygens) has more available reactive oxygen sites than TrimetaP (3 oxygens)^36^ (Fig. 9). Although HexametaP (6) has more reactive oxygen sites on a molar basis than TripolyP, at equal mass concentrations, as in our study, TripolyP would supply a greater number of reactive oxygen sites, resulting in the higher lead concentrations observed in the CTSR. Secondly, while TrimetaP would be more ionized than TripolyP^12^, the greater flexibility (relative freedom of molecules to move, entangle, and disentangle) of TripolyP more easily allows metal ions to be fitted into its structure while cyclophosphates are sterically inhibited from assuming all possible configurations, reducing their interaction with metal ions^15^. Furthermore, longer chains allow for greater flexibility in the polymer bonds and metal incorporation into the polyphosphate structure^15^. The increased lead release with HexametaP compared to TrimetaP may be due to a combination of decreased ring strain and a greater number of reactive oxygen sites.

**Figure 6:**
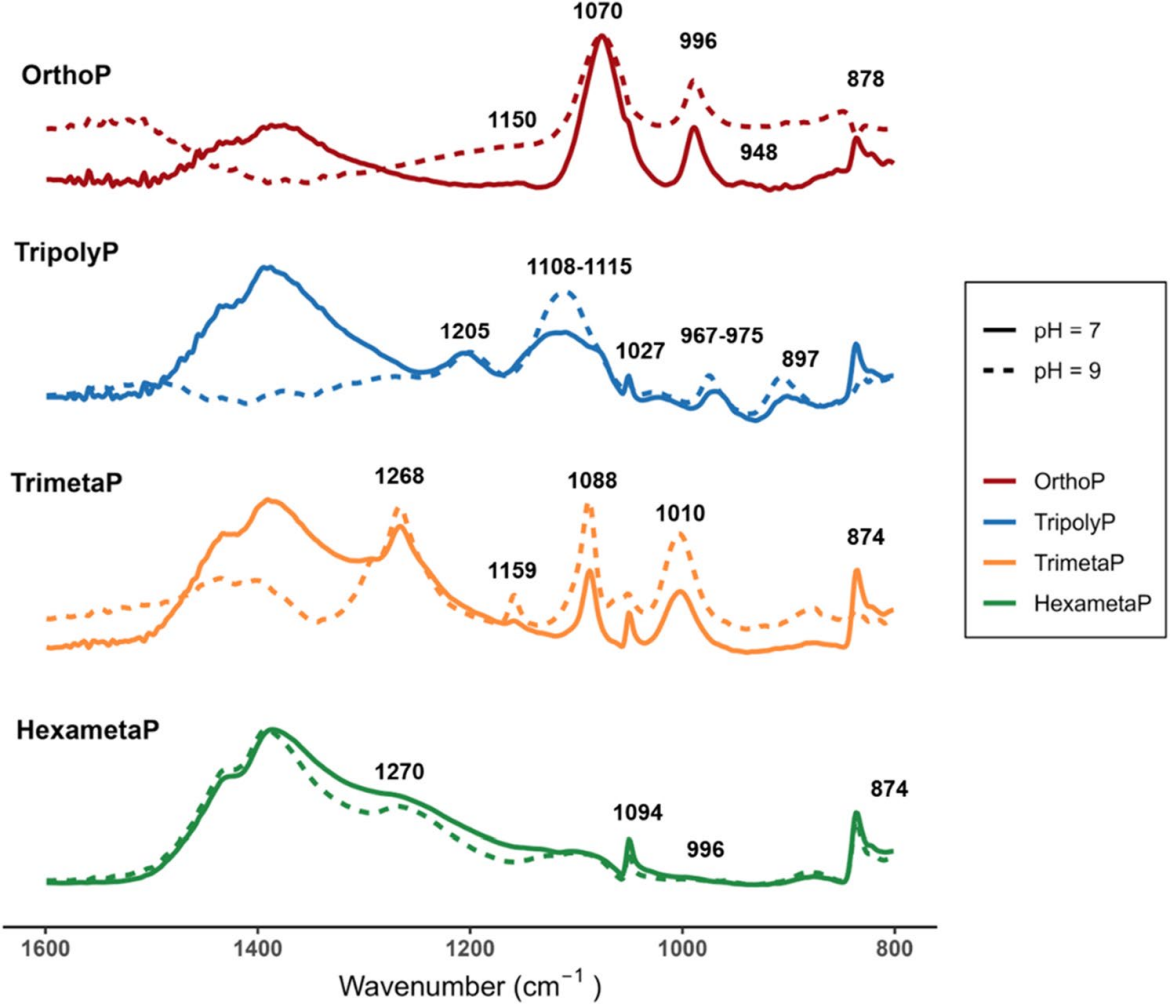
ATR-FTIR spectra of adsorbed phosphates as a function of pH (7.0 or 9.0) and phosphate type (OrthoP, TripolyP, TrimetaP, HexametaP) at DIC 5 mg C L^−1^ from the batch experiment. All ATR-FTIR spectra were recorded in a DIC 5 mg C L^−1^ electrolyte solution*.*

The interaction between lead carbonate and TripolyP may be regulated by the formation of bidentate or tridentate complexes with ionized terminal PO_3_^−^ or when the middle phosphate group is also bound^37,38^. This is seen via the vibration bands at 1108–1115^31^ and 1205 cm^−1^^32^ respectively (Fig. 6). However, IR spectra suggest that not all phosphate groups were bound to the lead surface, as indicated by the presence of unbound P_2_O_7_ (967–975 cm^−1^)^35^ possibly allowing for further lead complexation.

TrimetaP bonds with lead via OPO_3_^−^ groups, seen in the increased vibration frequency at 1159 (v_s_ P–O) and 1268 cm^−1^ (v_as_ P–O)^34^. Steric conformation of cyclophosphate is suggested via the shift in vibration bands, indicating the lengthening and shortening of the P–O–P (902–874 cm^−1^) and P–O bonds (1002–1010 cm^−1^), respectively (Fig. 6). Compared to TrimetaP in solution, the vibration band at 1088 cm^−1^ (v_s_ P–O) did not shift positions suggesting that some phosphate groups in TrimetaP are sterically inhibited from interacting with the lead surface. Moreover, the intensity of the bands at 1008 (v_b_ P–O), 1086–1090 (v_s_ P–O), and 1268 cm^−1^ (v_as_ P–O) are larger in spectra representing TrimetaP than in those representing HexametaP, possibly caused by the binding of more phosphate groups per polyphosphate molecule with the lead carbonate surface (Fig. 6).

### Orthophosphate–polyphosphate blends

**Figure 7:**
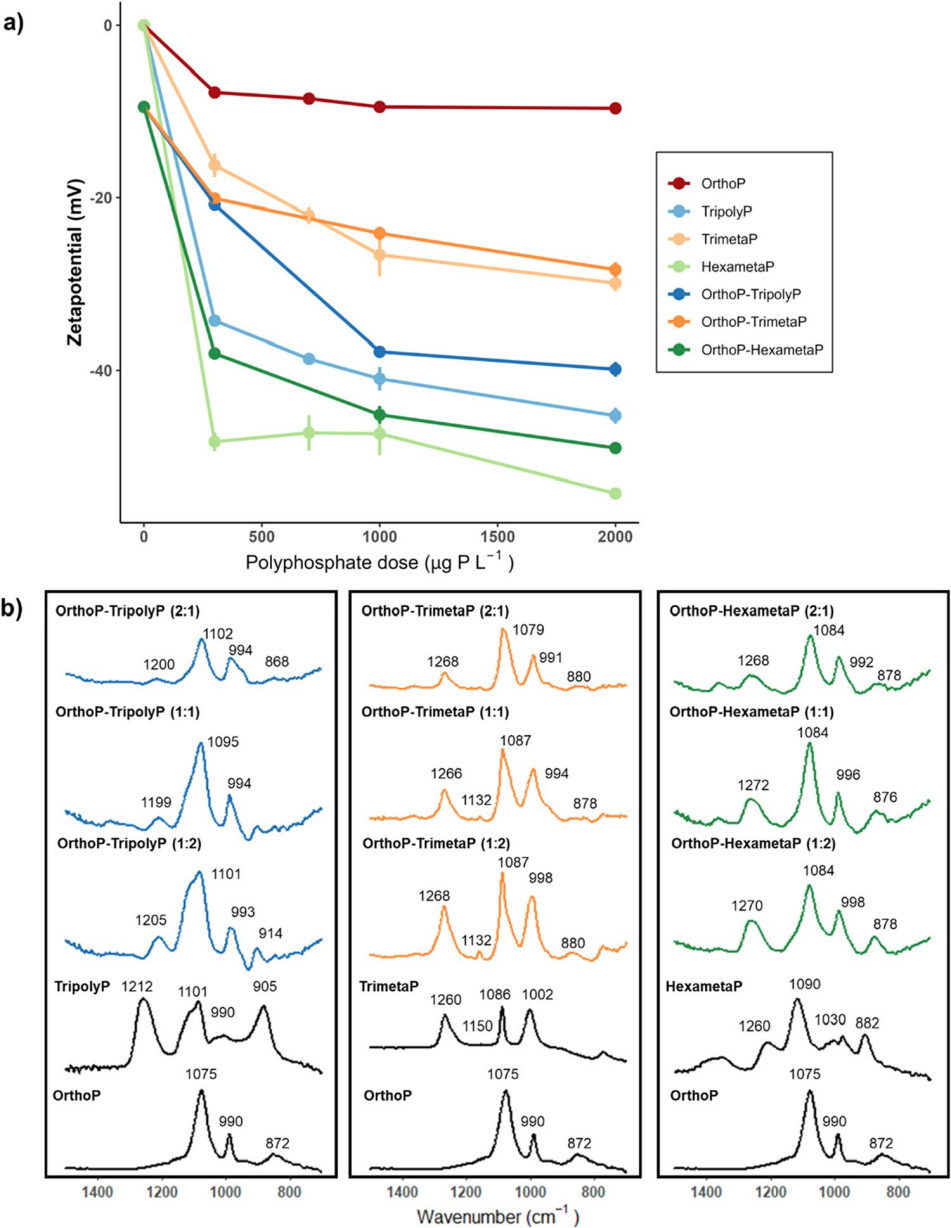
(**a**) Zetapotential and (**b**) ATR-FTIR spectra of adsorbed orthophosphate, polyphosphates, and orthophosphate–polyphosphates at pH 7.0. Orthophosphate:polyphosphate ratios are presented in brackets. Reference ATR-FTIR spectra of free orthophosphate and polyphosphate solutions are presented as solid black lines. All ATR-FTIR spectra were recorded in a DIC 5 mg C L^−1^ electrolyte solution.

Using mineral formation and lead dissolution results from the CSTR, along with the zeta potential data and ATR-FTIR spectra from batch experiments, we propose mechanisms for blended phosphate interactions with lead carbonate. First, the less pronounced charge reversal with orthophosphate–polyphosphates than polyphosphates alone, as well as the presence of IR peaks belonging to both adsorbed OrthoP and polyphosphates, suggests that both phosphate species simultaneously interact with the lead (II) carbonate (Fig. 7a,b). Second, the formation of lead-polyphosphate complexes is expected to keep free lead concentrations low, resulting in decreased hydroxypyromorphite formation and increased lead solubility.

**Figure 8:**
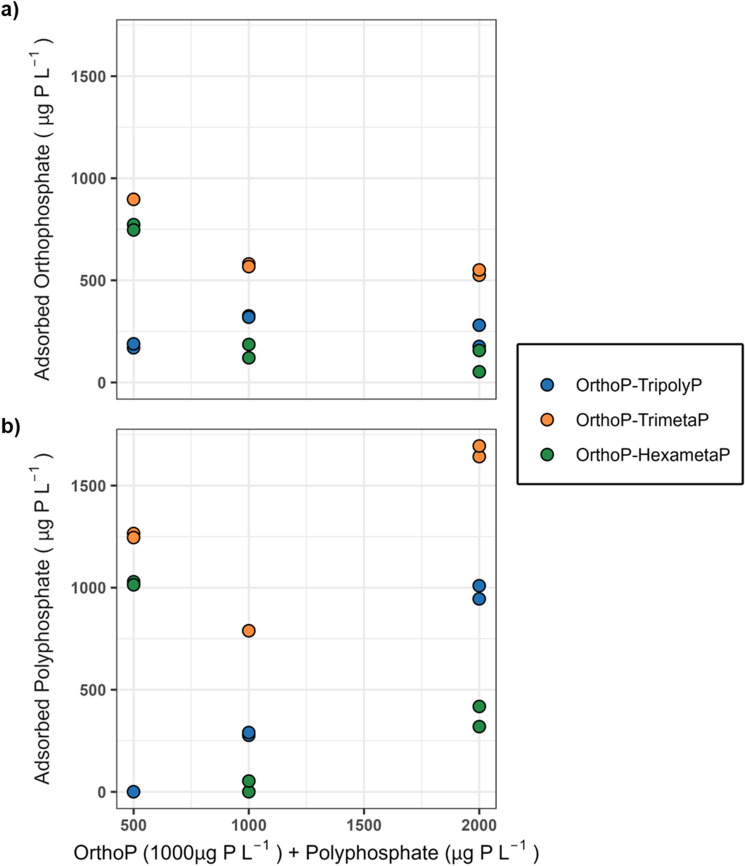
Loss of (**a**) orthophosphate and (**b**) polyphosphate at pH 7 from the batch reactors as a function of polyphosphate concentration (500–2000 µg P L^−1^). The solutions contained 1000 µg P L^−1^ orthophosphate and 5 mg C L^−1^ DIC.

FTIR data from the batch experiments suggest that polyphosphates interferes with OrthoP adsorption to the lead carbonate surface. This is supported by a comparison of orthophosphate and polyphosphate adsorption (Fig. 8a,b), quantified as the difference between the influent and effluent concentrations. Supplementary experiments (Sect. S3) showed that polyphosphate hydrolysis to OrthoP was expected to be less than 10% within the 30 min reaction time in solutions at pH 7.0 and a DIC of *5* mg C L^−1^. We can therefore attribute OrthoP (from phosphoric acid) as the dominant source of PO_4_ adsorbed after the 30 min reaction. Polyphosphate loss (adsorption + precipitation) to lead carbonate increased while—in the case of TrimetaP and HexametaP—OrthoP loss decreased with increasing polyphosphate concentrations.

IR spectra suggest that OrthoP (~ 850–888 and 990–998 cm^−1^) was adsorbed to lead (II) carbonate in all blends (Fig. 7b). In tests with cyclophosphate blends, increases in the peak intensities at 1260–1272 cm^−1^ are consistent with competitive adsorption of cylcophosphates. However, more TrimetaP than HexametaP or TripolyP was lost to the lead (II) carbonate (Fig. 8b). Moreover, the increase in peak intensities corresponding to TripolyP at 1095–1102 and 1199–1205 cm^−1^ as well as the disappearance of the OrthoP peak at 888 cm^−1^ suggests that more TripolyP than OrthoP was present on the lead (II) carbonate surface at the 1:2 ratio. This was supported by the 8.8 ± 2.5% decrease in the proportion of adsorbed phosphorus as OrthoP when TripolyP was increased from 1000 (1:1) to 2000 µg P L^−1^(1:2).

**Figure 9:**
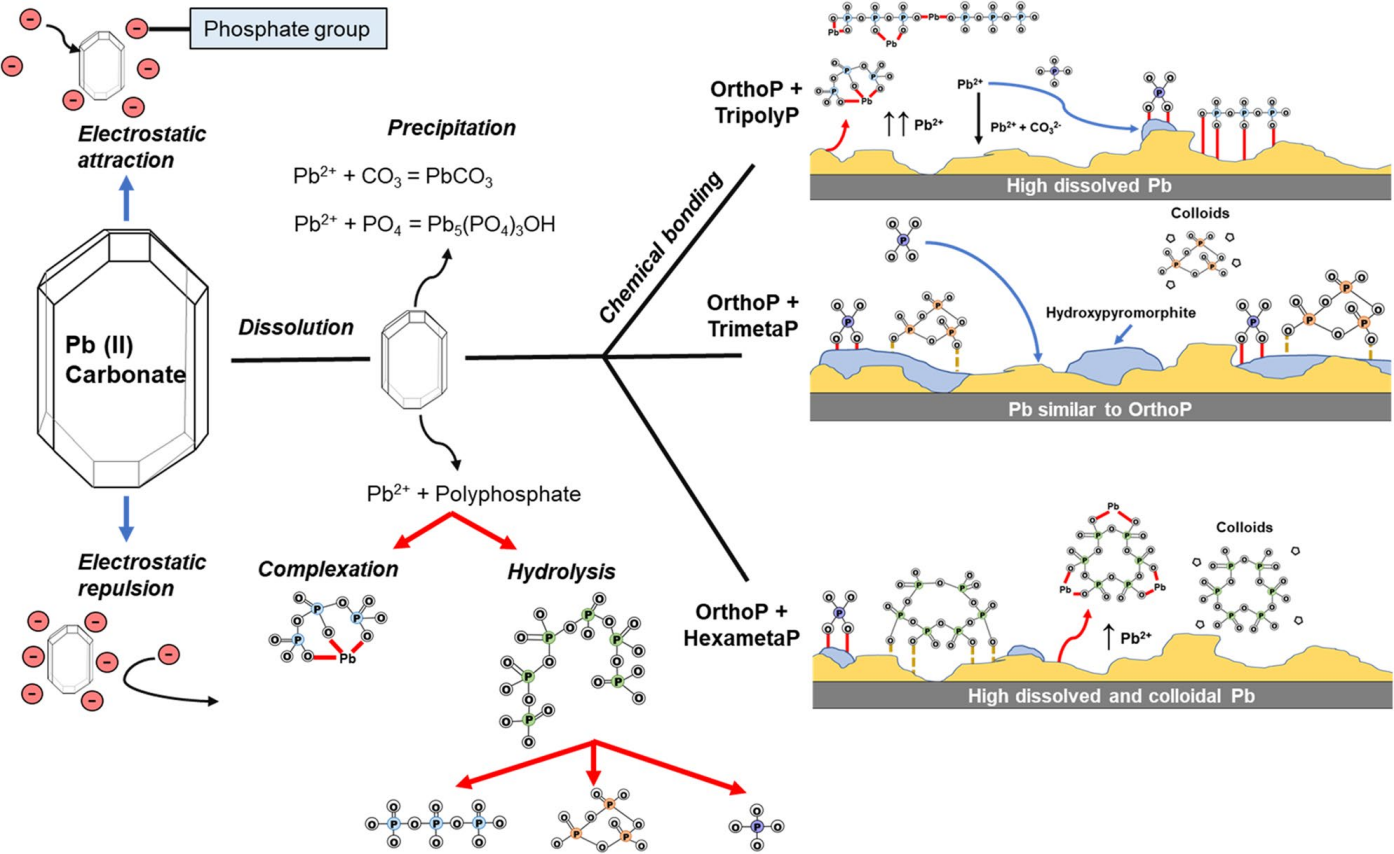
Schematic representation of phosphate-lead interaction mechanisms. Bonding information was sourced from Michelmore et al.^31^, and Rashchi and Finch^38^.

When lead (II) carbonate starts to dissolve, Pb^2+^ is released from the surface into aqueous solution. Pb^2+^ ions may either form complexes with polyphosphate, adsorb to the lead carbonate surface, or precipitate with other anions (e.g., PO_4_^3−^) at or away from the surface (Fig. 9). Precipitation may result in a different surface layer, possibly cerussite, hydrocerussite, or hydroxypyromorphite, forming. While OrthoP is expected to reduce lead release by forming hydroxypyromorphite, polyphosphates compete with OrthoP for lead binding sites. As binding sites on the lead (II) carbonate fill with adsorbed polyphosphates, vacant sites may be more difficult to access by free polyphosphates due to a combination of electric repulsion and structural interference from neighboring phosphate-filled sites. This was reflected by comparing the cyclophosphate blends: there was a − 20.7 ± 1.4 mV change in surface charge and a 10.7–30.8% decrease in adsorbed phosphorus between blends of OrthoP with TrimetaP than HexametaP. The amount of dissolved lead increased with polyphosphate concentration.

## Conclusion

Despite decades of use, significant knowledge gaps concerning corrosion control with blended phosphates remain. This study explored the effect of polyphosphate structure—in blended formulations with orthophosphate—on lead release and mineral formation, removing the uncertainties associated with proprietary blend formulations. We compared orthophosphate–polyphosphate blends made using either linear or cyclophosphates against orthophosphate alone, reaching the following conclusions.

1. TripolyP had greater lead-binding capacity than TrimetaP (1.00 vs 0.07 mol_Pb_/mol_polyphosphate_, respectively). The longer chain length cyclophosphate, HexametaP (1.60–2.10 mol_Pb_/mol_polyphosphate_), bound more lead than either TrimetaP or TripolyP.
2. Blending OrthoP and TrimetaP resulted in the lowest lead release among the orthophosphate–polyphosphate blends. In fact, blending trimetphosphate with orthophosphate had minimal impact on lead release compared to orthophosphate alone.
3. The application of TripolyP or HexametaP exacerbated the amount of dissolved and small colloidal lead. OrthoP-HexametaP treatment increased small colloid concentrations by 124.0 (119.4–131.7) and 980.0 (831.3–1102) µg Pb_0.2–0.45 µm_ m^−2^ at the 1:1 and 1:2 ratios, respectively and yielded a high proportion (26 and 49% for 1:1 and 1:2 ratio, respectively) at 24HS. Whereas OrthoP-TripolyP increased colloidal lead by 173.7 (144.9–190.8) µg Pb_0.2–0.45 µm_ m^−2^ only at the1:2 ratio. However, colloidal lead only accounted for 10% of total lead release at 24HS.
4. Polyphosphate inhibited formation of hydroxypyromorphite. Lead-polyphosphate complexes may have kept the solution undersaturated with respect to the lead minerals present, resulting in higher lead release. This effect was more pronounced at higher polyphosphate concentrations.

This study provides insight into the interactions and subsequent release of lead associated with blended phosphate treatment. These findings will help us better understand the conditions that might result in higher dissolved or colloidal lead as well as the formation of lead corrosion scale. OrthoP-TrimetaP blends may be a promising solution for simultaneous corrosion control and sequestration but further investigation on their ability to mitigate aesthetic water quality is needed.

## Supplementary Information

Supplementary Information.

## Data Availability

Experimental data are available at 10.5281/zenodo.6473898.
