# Supplementary material for: Impacts of orthophosphate–polyphosphate blends on the dissolution and transformation of lead (II) carbonate
Source: Sci Rep. 2022 Oct 25;12:17885. doi: 10.1038/s41598-022-22683-2 (PMC9596708; doi:10.1038/s41598-022-22683-2)
Supplement: Supplementary file 1 — Supplementary Information. [file 41598_2022_22683_MOESM1_ESM.docx]

SUPPORTING INFORMATION

Impacts of orthophosphate-polyphosphate blends on the dissolution and transformation of lead (II) carbonate

Javier A. Locsin^a,^*, Benjamin F. Trueman^a,b^, Evelyne Doré^a^, Aaron Bleasedale-Pollowy^a^, and Graham A. Gagnon^a^

^a^Centre for Water Resources Studies, Department of Civil & Resource Engineering, Dalhousie University, 1360 Barrington St., Halifax, Nova Scotia, Canada B3H 4R2
 
 ^b^Halifax Water, 450 Cowie Hill Road, Halifax, Nova Scotia, Canada B3P 2V3
 
  *Corresponding author: Javier Locsin
  E-mail: [javierlocsin@dal.ca](mailto:javierlocsin@dal.ca)
  Tel: 902.494.6070
  Fax: 902.494.3105

Supplementary Materials contains 22 pages with 3 sections, 5 figures, and 5 tables

**Table of contents**

[**Section S1.** ATR-FTIR of phosphates in solution 2](#_Toc116492819)

[**Section S2**. ATR-FTIR analysis of phosphates adsorbed on to lead carbonate 4](#_Toc116492820)

[**Section S3**. Polyphosphate hydrolysis to orthophosphate within 30 mins 8](#_Toc116492821)

[**Table S1:** Summary of conditions and results of the CSTR dissolution experiments 10](#_Toc116492822)

[**Table S2:** Summary of energy dispersive spectroscopy (EDS) results of CSTR solids. Data is presented in weight percent (wt. %) 12](#_Toc116492823)

[**Table S3:** Standard XRD patterns and their PDF entry numbers. 14](#_Toc116492824)

[**Table S4:** Standard ATR-FTIR patterns and their RUFF entry numbers. 15](#_Toc116492825)

[**Table S5:** Summary of polyphosphate hydrolysis experiments 16](#_Toc116492826)

[**Figure S1:** XRD sample and reference patterns for OrthoP-TripolyP experiments. 17](#_Toc116492827)

[**Figure S2:** XRD sample and reference patterns for OrthoP-TrimetaP experiments 18](#_Toc116492828)

[**Figure S3:** XRD sample and reference patterns for OrthoP-HexametaP experiments 19](#_Toc116492829)

[**Figure S4:** ATR-FTIR spectra of phosphate standards (1 g P L^-1^) in solution at pH 7 and 9. ATR-FTIR spectra were recorded in the 5 mg L^-1^ DIC background electrolyte solution. 20](#_Toc116492830)

[**Figure S5:** ATR-FTIR spectra of phosphates (1 g P L^-1^) adsorbed onto lead carbonate at pH 7 and 9. ATR-FTIR spectra were recorded in the 5 mg L^-1^ DIC background electrolyte solution. 21](#_Toc116492831)

[**References** 22](#_Toc116492832)

#

# Section S1. ATR-FTIR of phosphates in solution

A 50-mL volume of phosphate solution was prepared by adding 1 g P L^-1^ of hexametaphosphate (HexametaP, (NaPO_3_)_6_) (Alfa Aesar, Haverhill, MA), sodium trimetaphosphate (TrimetaP, (NaPO_3_)_3_) (Alfa Aesar, Haverhill, MA), sodium tripolyphosphate (TripolyP, Na_5_P_3_O_10_) (Alfa Aesar, Haverhill, MA), and orthophosphate (ACS grade phosphoric acid, Fisher Chemical, Fairlawn, NJ)) to ultrapure water (18.2 M$\Omega$cm, TOC < 2 $\mu$g L^-1^) with a dissolved organic carbon content of 5 mg C L^-1^. Dissolved organic carbon was achieved by dissolving sodium bicarbonate powder (Fisher Chemical, Fairlawn, NJ). The pH, measured on an Acument XL50, was adjusted to either pH 7 or 9 by the addition of 1N trace metal grade nitric acid (Fisher Chemical, Fairlawn, NJ) or freshly prepared 2N sodium hydroxide (Fisher Chemical, Fairlawn, NJ).

For analyzing the phosphate solutions, a single-beam Fourier transform infrared spectroscopy in attenuated total reflectance mode (ATR-FTIR) (Bruker alpha-P, USA) was used. After pH adjustment, 20 $\mu$L of sample was deposited onto the ATR crystal using a pipette and analyzed. Each ATR spectrum was recorded with the blank cell as the background. Fifty scans at a wavenumber range between 400-4000 cm^-1^ were co-added to obtain each spectrum, with a resolution of 4 cm^-1^. Baseline spectra for ultrapure water with 5 mg C L^-1^ at pH 7 or 9 was measured in a similar way. Spectral subtraction of the IR spectra of baseline water from the sample spectra produced the spectra of dissolved phosphate species (Figure S4).

The peak assignments for the ATR-FTIR spectra of dissolved phosphates were based on the data of condensed phosphates (Guan et al., 2005; Lu et al., 2019; Michelmore et al., 2000; Socrates, 2004). The important features are summarized in table S3.

# Section S2. ATR-FTIR analysis of phosphates adsorbed on to lead carbonate

2.1 Sample preparation and collection

A 50-mL suspension of 1 g L^-1^ lead (II) carbonate (Alfa Aesar, Haverhill, MA) was prepared by dissolving sodium bicarbonate powder (Fisher Chemical, Fairlawn, NJ) in ultrapure water (18.2 M$\Omega$cm, TOC < 2 $\mu$g L^-1^) to achieve a 5 mg C L^-1^ dissolved inorganic concentration then adding lead (II) carbonate powder. Phosphates were added at 1 g P L^-1^ as either hexametaphosphate (HexametaP, (NaPO_3_)_6_) (Alfa Aesar, Haverhill, MA), sodium trimetaphosphate (TrimetaP, (NaPO_3_)_3_) (Alfa Aesar, Haverhill, MA), sodium tripolyphosphate (TripolyP, Na_5_P_3_O_10_) (Alfa Aesar, Haverhill, MA), and orthophosphate (ACS grade phosphoric acid, Fisher Chemical, Fairlawn, NJ)). The pH, measured on an Acument XL50, was adjusted to either pH 7 or 9 by the addition of 1N trace metal grade nitric acid (Fisher Chemical, Fairlawn, NJ) or freshly prepared 2N sodium hydroxide (Fisher Chemical, Fairlawn, NJ). The suspensions were placed on a shaker table for 30-mins at 150 rpm. At the end of the reaction period, samples were analyzed with a single-beam Fourier transform infrared spectroscopy in attenuated total reflectance mode (ATR-FTIR) (Bruker alpha-P, USA).

A thin layer of lead (II) carbonate powder from the reactors was deposited onto the ATR crystal using a plastic spoon and analyzed. Each ATR spectrum was recorded with the blank cell as the background. Fifty scans at a wavenumber range between 400-4000 cm^-1^ were co-added to obtain each spectrum, with a resolution of 4 cm^-1^. Baseline spectra for lead (II) carbonate at pH 7 or 9 was obtained by adding 1 g L^-1^ of powder to ultrapure water with 5 mg C L^-1^ and mixing for 30-mins at 150 rpm. Spectral subtraction of the IR spectra of baseline suspension from the sample spectra produced the spectra of adsorbed phosphate species.

2.2 Sample analysis

The peak assignments for the ATR-FTIR spectra of adsorbed phosphates on lead carbonate are based on the data of condensed phosphates adsorption on titania and metal (hydr)oxides (Guan et al., 2005; Lu et al., 2019; Michelmore et al., 2000; Socrates, 2004). The important features are summarized in table S1. Due to the complexity caused by the solubility of Pb (II) carbonate at these conditions, carbonate bands representative of cerussite were detected at 670 and 835 cm^-1^ in all experiments. This may indicate surface precipitation of Pb carbonate species or be due to the incomplete surface coverage of phosphate on the lead surface.

The vibration band positions of orthophosphate adsorbed onto Pb (II) carbonate were fairly consistent across pH 7 and 9. Orthophosphate at pH 7 contains a mixture of both H_2_PO_4_^-^ and HPO_4_^2-^, with bands for both species observed. Vibration bands corresponding to H_2_PO_4_^-^ at 878, 948, 1070, and 1150 cm^-1^ were present. Vibration bands corresponding to HPO_4_^2-^ at 855 and 940 cm^-1^ were present. The bands at 878 and 948 cm^-1^, and 1070 and 1150 cm^-1^ are assigned to the symmetric (v_s_) and asymmetric (v_as_) stretching vibration of P-O-P and P-O, respectively. The decreased frequency of the 1077 and 1157 cm^-1^ bands to 1070 and 1150 cm^-1^ indicated a weakening of the P-O bond. While the increased frequency of the 872 and 940 cm^-1^ bands to 878 and 946 cm^-1^ indicated the formation of a P-O-Pb bond. The weak bands at 1070 and 1110 cm^-1^, and the shift to higher frequencies of the 875 and 944 cm^-1^ bands were attributed to the weakening of the P-O bond during adsorption as well as the stronger P-O-Pb bond compared to P-OH bonds in solution. Increased pH resulted in similar adsorbed phosphate species, but with increased intensities in the vibration bands corresponding to HPO_4_^2-^. The presence of outer sphere, electrostatically adsorbed phosphate (1007 cm^-1^) was not detected (Figure S5).

Tripolyphosphate adsorption onto Pb exhibited minimal change in the position of phosphate vibration bands across both pH, indicating that the adsorbed species is independent of pH (Figure S4). In the FTIR spectra of adsorbed tripolyphosphate, six peaks dominate the spectra at both pH: 897, 967-975, 1027, 1052, 1108-1115, 1205 cm^-1^. These were assigned to the v_as_(P-O-P), v_as_(P_2_O_7_^-^), v_as_(P-OH), v_as_(terminal PO_3_^-^), and v_as_(bridging PO_2_). We assigned the bands at 1108-1115 cm^-1^ to v_as_(terminal PO_3_^-^) by the comparison of the adsorption bands of orthophosphate to metal oxides (Guan et al., 2005; Tejedor-Tejedor and Anderson, 1990). The 1108-1115 cm^-1^ band fell between the frequency of v_as_(P-O) bond in H_2_PO_4_^-^ and v_s_(P-O) bond in HPO_4_^2-^, which were assigned to the v_as_(P-O) bond in the bidentate complexes formed between the terminal PO_3_^-^ and the lead carbonate surface. After the reaction with lead, the appearance of the 967-975 cm^-1^ bands, attributed to unbound P_2_O_7_, suggested that not all phosphate groups were bound to the Pb surface (Wan et al., 2020). Additionally, at higher pH, the increased intensity of the bands at 897, 975, and 1115 cm^-1^ at the expense of the band at 1050 cm^-1^ reflected the increased interaction of terminal PO_3_^-^ groups.

The FTIR spectra presented by adsorbed trimetaphosphate or hexametaphosphate were similar across pH (Figure S5). Five distinct peaks dominated trimetaphosphate spectra: bands at 874, 1010, 1088, 1159, and 1268 cm^-1^ were assigned to v_s_(P-O-P), bending vibration v_b_(P-O), v_s_(P-O), and v_as_(P-O). The bands at 1159 and 1268 cm^-1^ suggested the formation of a P-O-Pb bond. Furthermore, when compared to trimetaphosphate in solution, the lengthening of the P-O-P and shortening of the P-O bonds in the adsorbed species is observed via the shift of the bands from 902 to 874 cm ^-1^ and 1002 to 1010 cm^-1^, respectively. This suggested the steric conformation of trimetaphosphate on the Pb surface.

Only four distinct peaks were attributed to phosphate species with adsorbed hexametaphosphate: the bands at 874, 996, 1094, and 1270 cm^-1^ were assigned to v_as_(P-O-P), v_b_(P-O), and v_as_(P-O), respectively (Figure S5). Similarly, the lengthening of the v_as_(P-O-P) and shortening of the v_b_(P-O) bonds indicated steric conformation of hexametaphosphate on the lead surface. Whereas, the shift from 1086 and 1260 to 1094 and 1270 cm^-1^ indicated the depolymerization of hexametaphosphate into shorter chains and the formation of Pb-phosphate structural units (Jha et al., 2015). The shift to lower frequency of the symmetrical P-O-P band at 874 cm^-1^ implied that some of the phosphate groups were not associated with the Pb surface. Moreover, the intensity of the bands at 1008 ,1086, and 1268 cm^-1^ are larger with trimetaphosphate than hexametaphosphate, possibly caused by the binding of more phosphate groups per polyphosphate molecule.

# Section S3. Polyphosphate hydrolysis to orthophosphate within 30 mins

3.1 Experimental design and sample analysis

Polyphosphate hydrolysis experiments were initiated by adding 1 mg Pb L^-1^ PbNO_3_ (Fisher Chemical, Fairlawn, NJ) in 100 mL of phosphate solution. Sodium bicarbonate powder (Fisher Chemical, Fairlawn, NJ) was dissolved in ultrapure water (18.2 M$\Omega$cm, TOC < 2 $\mu$g L^-1^) to achieve a 5 mg C L^-1^ dissolved inorganic concentration. Polyphosphates were added at 300 or 1000 µg P L^-1^ as either hexametaphosphate (HexametaP, (NaPO_3_)_6_) (Alfa Aesar, Haverhill, MA), sodium trimetaphosphate (TrimetaP, (NaPO_3_)_3_) (Alfa Aesar, Haverhill, MA), and sodium tripolyphosphate (TripolyP, Na_5_P_3_O_10_) (Alfa Aesar, Haverhill, MA). For blended phosphate experiments, 300 µg P L^-1^ of HexametaP, TrimetaP, or TripolyP were combined with 300 µg P L^-1^ orthophosphate (ACS grade phosphoric acid, Fisher Chemical, Fairlawn, NJ)). The pH, measured on an Acument XL50, was adjusted to either pH 7.5 ± 0.2 by the addition of 1N trace metal grade nitric acid (Fisher Chemical, Fairlawn, NJ) or freshly prepared 1N sodium hydroxide (Fisher Chemical, Fairlawn, NJ). The suspensions were placed on a shaker table for 30-mins at 150 rpm. Prior to the addition of Pb and at the end of the 30 min reaction period, samples were analyzed for orthophosphate (PO_4_) via a HACH DR5000 (HACH, CO, USA) using the PhosVer 3 (#8048) method. The detection range for orthophosphate is 0.02 to 2.5 mg/L. Due to the requirement for near-instantaneous PO_4_ measurements, the HACH method was chosen over the more sensitive, but longer, ion chromatography method. All experiments were run in triplicate at room temperature (21 ± 2°C).

3.2 Results and discussion

Table S5 shows the amount of polyphosphate hydrolysis to orthophosphate at the end of a 30-min reaction time. Results show that hydrolysis followed TripolyP (6.5%) > HexametaP (3.4%) > TrimetaP (1.8%) at equivalent phosphorous to lead ion concentrations. When orthophosphate (300 µg P L^-1^) and polyphosphate (300 µg P L^-1^) were blended with 1000 µg Pb L^-1^, hydrolysis followed a similar pattern as above: Blends with TripolyP, TrimetaP, or HexametaP presented 9.4, 6.5, and 7.6% hydrolysis to orthophosphate. The 1000 µg Pb L^-1^ concentration was higher than what was seen in the reactors with blended phosphates but was chosen to promote Pb-ion facilitated hydrolysis.

**Table S1:** Summary of conditions and results of the CSTR dissolution experiments

| ID | HRT = 30 min | | | | | HRT = 24 h | | | | |
| --- | --- | --- | --- | --- | --- | --- | --- | --- | --- | --- |
|  | Lead dissolution (µg Pb m^-2^) | | Phosphorous and phosphate removed in the reaction | | Dissolved lead in effluent (µmol) | Lead (µg Pb m^-2^) | | Phosphorus and phosphate removed in the reaction | | Dissolved lead in effluent (µmol) |
|  | Dissolved (<0.2 µm) | <0.45 µm filtrate | Phosphorus (%) | Phosphate (%) |  | Dissolved (<0.2 µm) | <0.45 µm filtrate | Phosphorous (%) | Phosphate (%) |  |
| Phosphate free | 104.4 ± 4.3 | 118.6 ± 12.1 | * | * | 0.39 ± 0.02 | 168.4 ± 10.6 | 344.3 ± 13.1 | * | * | 0.64 ± 0.04 |
| OrthoP (300 µg P L^-1^) (Ref) | 65.7 ± 16.1 | 77.1 ± 10.2 | 3.0 ± 1.3 | * | 0.25 ± 0.06 | 97.1 ± 3.1 | 108.0 ± 6.3 | 4.1 ± 0.2 | * | 0.37 ± 0.01 |
| OrthoP (1000 µg P L^-1^) | 6.1 ± 2 | 19.2 ± 3.9 | 0.9 ± 0.7 | * | 0.02 ± 0.01 | 28.5 ± 2.8 | 98.7 ± 11.3 | 16.3 ± 4.9 | * | 0.11 ± 0.01 |
| OrthoP (Ref) + TrimetaP (300 µg P L^-1^) | 71.9 ± 5.7 | 75.1 ± 6.8 | 66.8 ± 0.7 | 97.9 ± 1.3 | 0.27 ± 0.02 | 95.6 ± 4.6 | 108.5 ± 10.6 | 67.7 ± 0.2 | 87 ± 32. | 0.36 ± 0.02 |
| OrthoP (Ref) + TrimetaP (700 µg P L^-1^) | 68.5 ± 1.1 | 75.2 ± 6.2 | 25.2 ± 1.1 | 98.2 ± 1.1 | 0.26 ± 0.004 | 116.0 ± 2.0 | 136.0 ± 8.1 | 29.8 ± 0.2 | 87.3 ± 32.7 | 0.44 ± 0.01 |
| OrthoP (Ref) + HexametaP (300 µg P L^-1^) | 50.2 ± 11.6 | 52.2 ± 11.6 | 1.3 ± 1.8 | 8.34 ± 3.1 | 0.19 ± 0.03 | 354.3 ± 14.9 | 480.1 ± 24.5 | 63.7 ± 1.7 | 7.4 ± 2.9 | 1.34 ± 0.06 |
| OrthoP (Ref) + HexametaP (700 µg P L^-1^) | 1165.0 ± 113.0 | 1742 ± 92.2 | 6.0 ± 4.0 | 10.2 ± 1.6 | 6.23 ± 0.43 | 1144 ± 125.3 | 2251.9 ± 63.6 | 33.3 ± 1.2 | 9.1 ± 3.7 | 4.31 ± 0.47 |
| OrthoP (Ref) + TripolyP (300 µg P L^-1^) | 614.8 ± 12.3 | 658.1 ± 21.4 | 19.2 ± 6.4 | 12.6 ± 2.6 | 2.31 ± 0.05 | 311.4 ± 28.1 | 347.9 ± 33.4 | 13.8 ± 4.6 | 11.2 ± 4.8 | 1.17 ± 0.11 |
| OrthoP (Ref) + TripolyP (700 µg P L^-1^) | 2052.6 ± 34.2 | 2048.7 ± 46.7 | 3.4 ± 1.4 | 0 ± 4.0 | 7.73 ± 0.13 | 1550.1 ± 5.5 | 1729.5 ± 28.5 | 18.7 ± 9.9 | 0 ± 4.2 | 5.84 ± 0.02 |
| TrimetaP (1000 µg P L^-1^) | 188.6 ± 23.3 | 265.1 ± 12.6 | 3.1 ± 0.9 | * | 0.71 ± 0.02 | 167.7 ± 6.5 | 340.9 ± 31.8 | 8.3 ± 0.7 | * | 0.63 ± 0.02 |
| HexametaP (1000 µg P L^-1^) | 2333.3 ± 156.1 | 2503.8 ± 211.9 | 6.6 ± 5.0 | * | 6.0 ± 0.09 | 2942.9 ± 111.2 | 2970.5 ± 112.1 | 7.7 ± 3 | * | 7.01 ±0.16 |
| TripolyP (1000 µg P L^-1^) | 2906.4 ± 27.6 | 2969.9 ± 72.2 | 0.5 ± 2.8 | * | 11.0 ± 0.10 | 2907.7 ± 20.9 | 3008.3 ± 21.9 | 8.8 ± 3.7 | * | 11.04 ± 0.00 |

*Data not available

**Table S2:** Summary of energy dispersive spectroscopy (EDS) results of CSTR solids. Data is presented in weight percent (wt. %)

| ID | Orthophosphate | Trimetaphosphate | Hexametaphosphate | Tripolyphosphate | P | Pb | C | O |
| --- | --- | --- | --- | --- | --- | --- | --- | --- |
|  | µg P L^-1^ | | | | wt.% (mean ± std dev) | | | |
| OrthoP (Ref) + TrimetaP (300 µg P L^-1^) | 300 | 300 | 0 | 0 | 25.82 ± 7.26 | 24.05 ± 6.11 | 17.16 ±8.82 | 32.94 ± 9.39 |
| OrthoP (Ref) + TrimetaP (700 µg P L^-1^) | 300 | 700 | 0 | 0 | 6.44 ± 2.42 | 65.84 ± 6.74 | 8.87 ±4.57 | 20.65 ± 4.64 |
| OrthoP (Ref) + HexametaP (300 µg P L^-1^) | 300 | 0 | 300 | 0 | 13.79 ± 3.46 | 57.64 ± 8.45 | 12.24 ± 6.04 | 16.34 ± 1.51 |
| OrthoP (Ref) + HexametaP (700 µg P L^-1^) | 300 | 0 | 700 | 0 | 0.24 ± 0.11 | 73.34 ± 6.36 | 7.63 ± 2.49 | 18.92 ± 4.29 |
| OrthoP (Ref) + TriployP (300 µg P L^-1^) | 300 | 0 | 0 | 300 | 41.69 ± 3.74 | 46.92 ± 9.96 | 3.75 ± 2.01 | 7.71 ± 4.48 |
| OrthoP (Ref) + TriployP (700 µg P L^-1^) | 300 | 0 | 0 | 700 | 1.55 ± 1.41 | 68.55 ± 4.56 | 10.8 ± 3.21 | 19.10 ± 8.84 |

**Table S3:** Standard XRD patterns and their PDF entry numbers.

| Phase | PDF entry No. |
| --- | --- |
| Hydroxypyromorphite | 00-089-6287 |
| Hydrocerussite | 96-901-1389 |
| Cerussite | 00-076-2056 |

**Table S4:** Standard ATR-FTIR patterns and their RUFF entry numbers.

| Phase | RUFF entry No. |
| --- | --- |
| Hydroxypyromorphite | NA |
| Hydrocerussite | R160062 |
| Cerussite | R040069 |

**Table S5:** Summary of polyphosphate hydrolysis experiments

| ID | OrthoP | | TrimetaP | HexametaP | TripolyP | | Lead | Initial PO_4_ | PO_4_ at end of 30-min HRT | Hydrolysis (%) |
| --- | --- | --- | --- | --- | --- | --- | --- | --- | --- | --- |
|  | µg P L^-1^ | | | | | | µg Pb L^-1^ | mg PO_4_ L^-1^ | mg PO_4_ L^-1^ |  |
| TripolyP (1000 µg P L^-1^) | 0 | 0 | | 0 | | 1000 | 1000 | 0.01 | 0.21±0.01 | 6.50±0.30 |
| TrimetaP (1000 µg P L^-1^) | 0 | 1000 | | 0 | | 0 | 1000 | 0.01 | 0.06±0.02 | 1.80±0.80 |
| HexametaP (1000 µg P L^-1^) | 0 | 0 | | 1000 | | 0 | 1000 | 0.01 | 0.11±0.01 | 3.40±0.40 |
| OrthoP (Ref) + TripolyP (300 µg P L^-1^) | 300 | 0 | | 0 | | 300 | 1000 | 0.94±0.01 | 1.03±0.01 | 9.40±0.50 |
| OrthoP (Ref) + TrimetaP (300 µg P L^-1^) | 300 | 300 | | 0 | | 0 | 1000 | 0.94±0.01 | 0.99±0.02 | 6.50±1.50 |
| OrthoP (Ref) + HexametaP (300 µg P L^-1^) | 300 | 0 | | 300 | | 0 | 1000 | 0.94±0.01 | 1.01±0.02 | 7.20±0.50 |

Note: values is red are below detection limit and were assigned half the detection limit value (0.01 mg PO_4_ L^-1^)


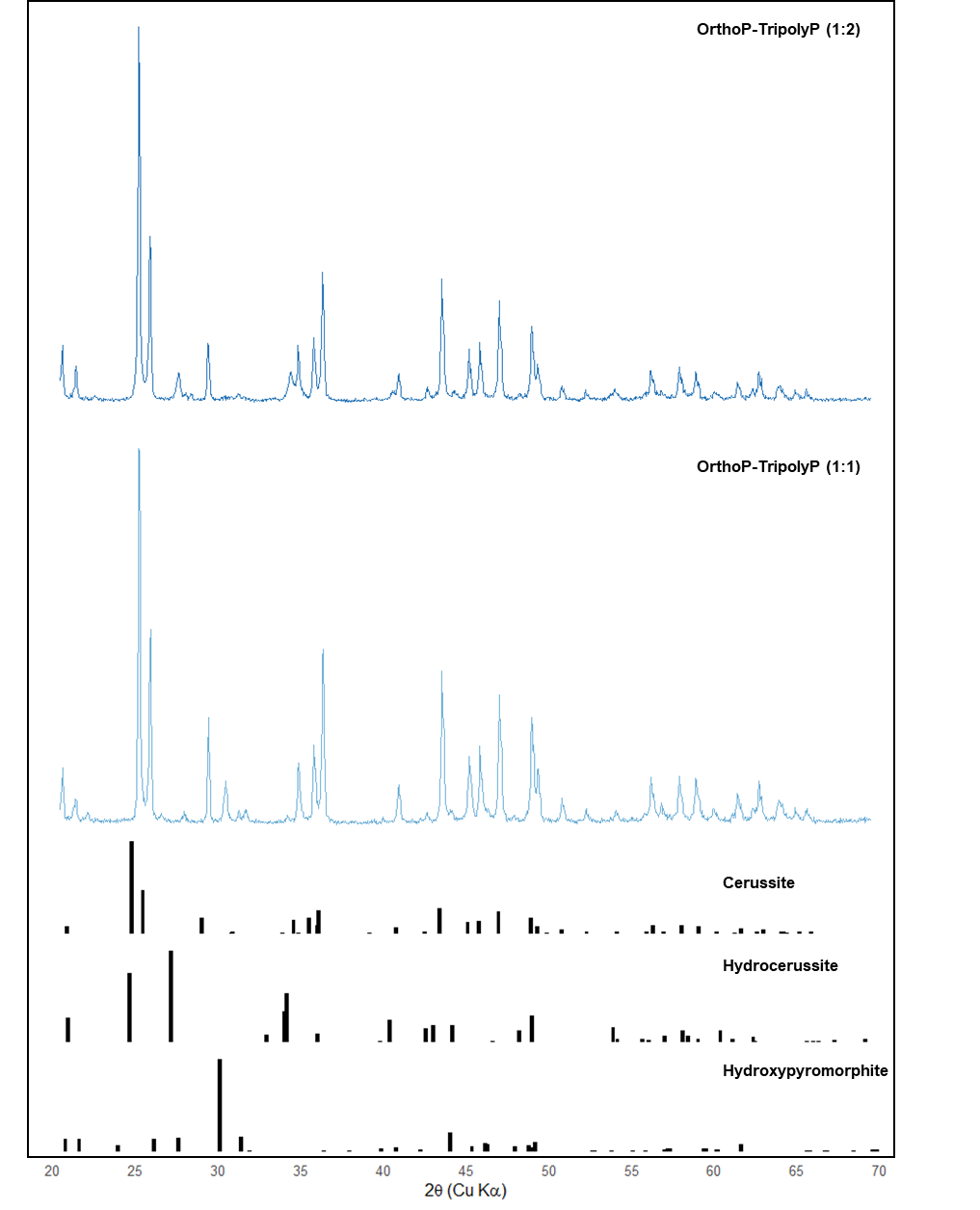


**Figure S1:** XRD sample and reference patterns for OrthoP-TripolyP experiments.


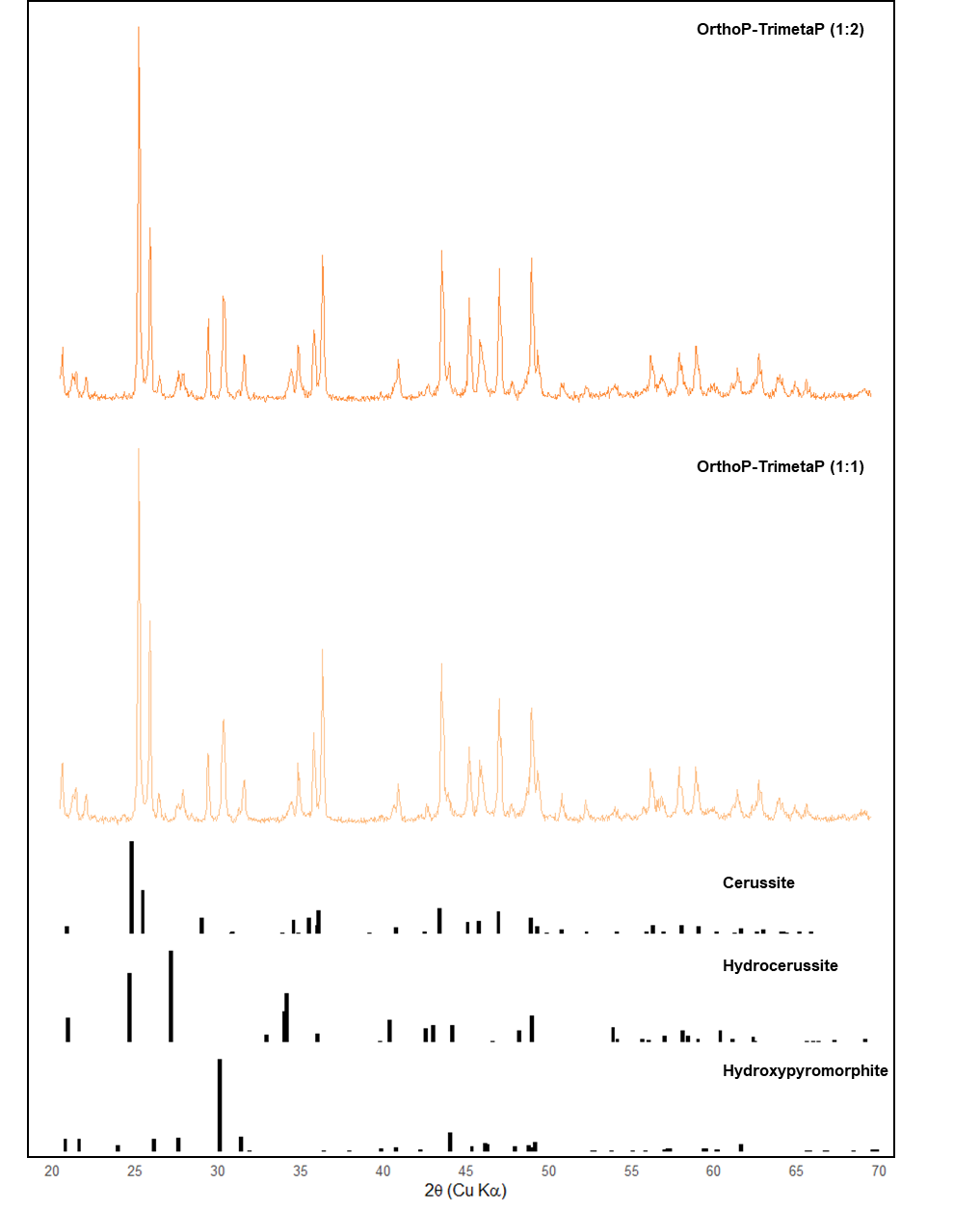


**Figure S2:** XRD sample and reference patterns for OrthoP-TrimetaP experiments


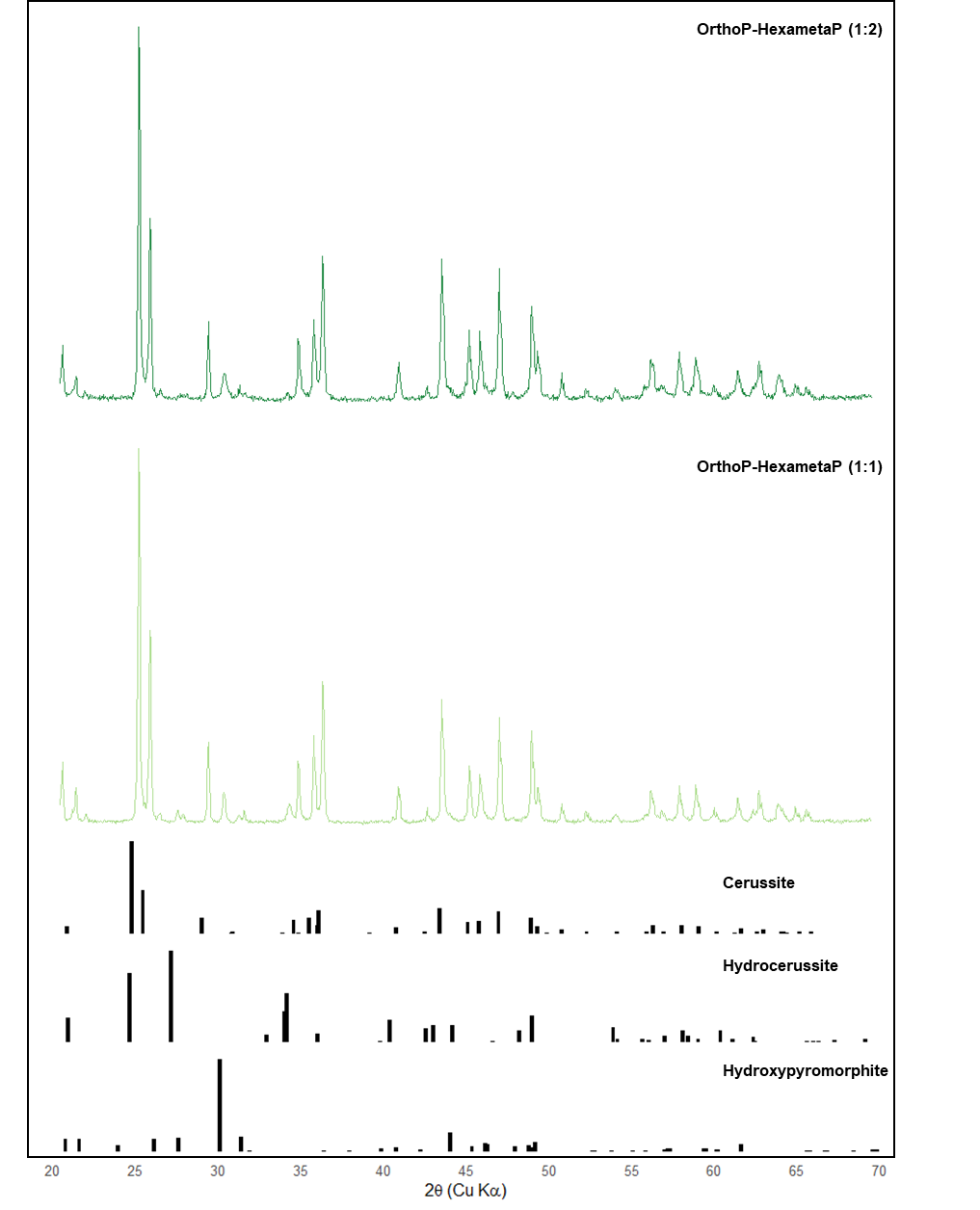


**Figure S3:** XRD sample and reference patterns for OrthoP-HexametaP experiments


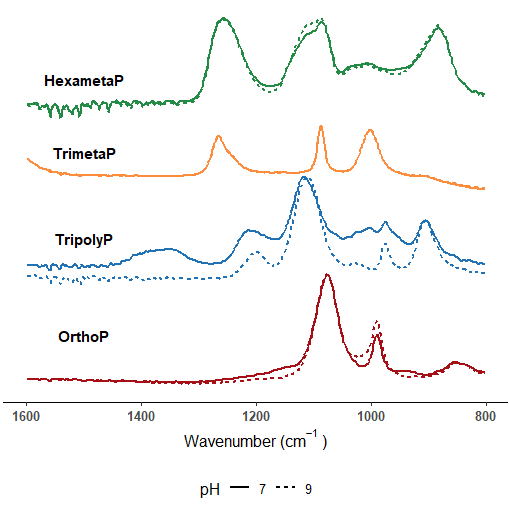


**Figure S4:** ATR-FTIR spectra of phosphate standards (1 g P L^-1^) in solution at pH 7 and 9. ATR-FTIR spectra were recorded in the 5 mg L^-1^ DIC background electrolyte solution.


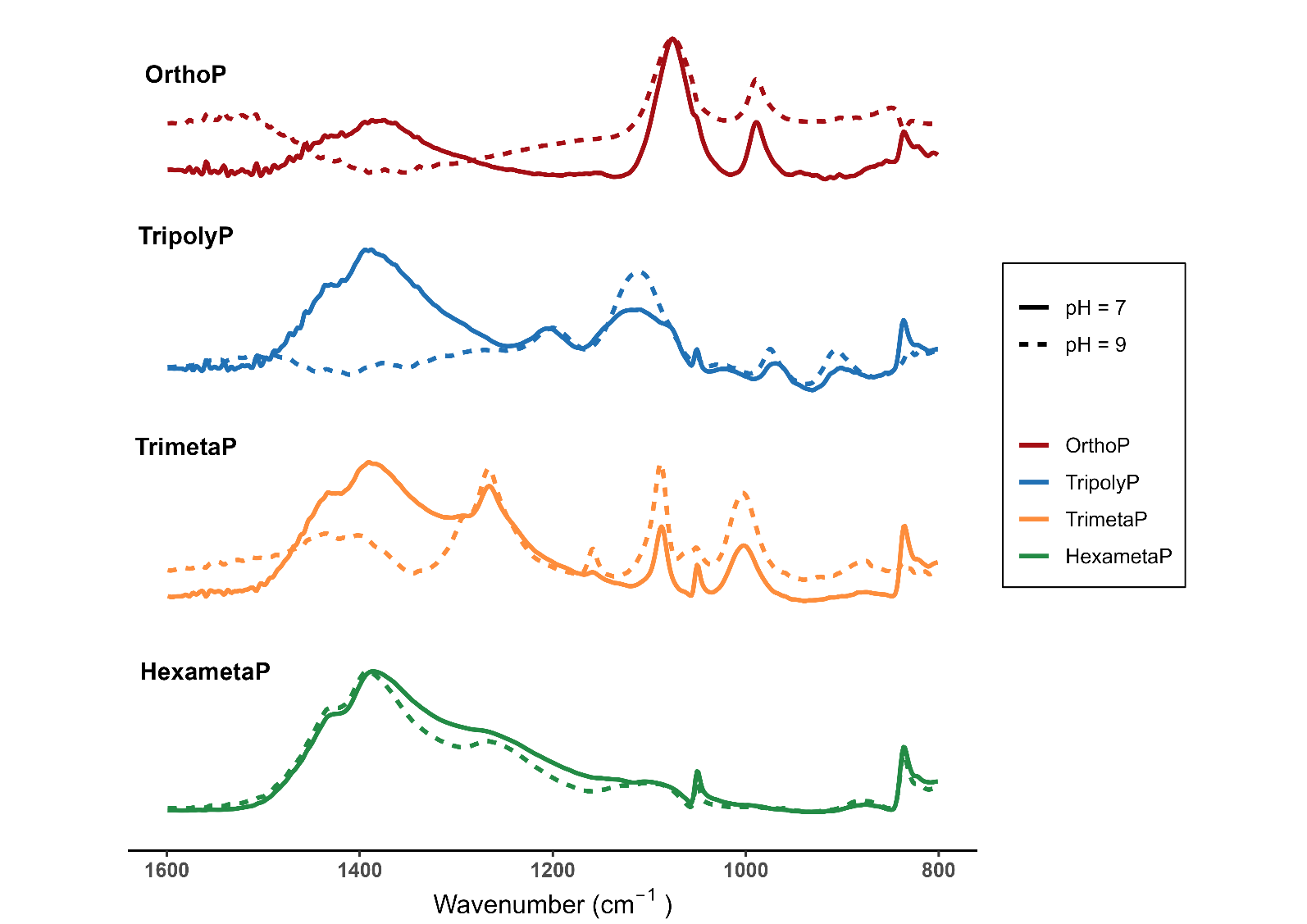


**Figure S5:** ATR-FTIR spectra of phosphates (1 g P L^-1^) adsorbed onto lead carbonate at pH 7 and 9. ATR-FTIR spectra were recorded in the 5 mg L^-1^ DIC background electrolyte solution.

**References**

Guan, X.-H., Liu, Q., Chen, G.-H., Shang, C., 2005. Surface complexation of condensed phosphate to aluminum hydroxide: An ATR-FTIR spectroscopic investigation. Journal of colloid and interface science 289, 319–327. <https://doi.org/10.1016/j.jcis.2004.08.041>

Jha, P.K., Pandey, O., Singh, K., 2015. FTIR spectral analysis and mechanical properties of sodium phosphate glass–ceramics. Journal of Molecular Structure 1083, 278–285. <https://doi.org/10.1016/j.molstruc.2014.11.027>

Lu, J., Sun, M., Yuan, Z., Qi, S., Tong, Z., Li, L., Meng, Q., 2019. Innovative insight for sodium hexametaphosphate interaction with serpentine. Colloids and Surfaces A: Physicochemical and Engineering Aspects 560, 35–41. <https://doi.org/10.1016/j.colsurfa.2018.09.076>

Michelmore, A., Gong, W., Jenkins, P., Ralston, J., 2000. The interaction of linear polyphosphates with titanium dioxide surfaces. Physical Chemistry Chemical Physics 2, 2985–2992. <https://doi.org/10.1039/B001213K>

Socrates, G., 2004. Infrared and raman characteristic group frequencies: Tables and charts. John Wiley & Sons.

Tejedor-Tejedor, M.I., Anderson, M.A., 1990. The protonation of phosphate on the surface of goethite as studied by CIR-FTIR and electrophoretic mobility. Langmuir 6, 602–611. <https://doi.org/10.1021/la00093a015>

Wan, B., Elzinga, E.J., Huang, R., Tang, Y., 2020. Molecular mechanism of linear polyphosphate adsorption on iron and aluminum oxides. The Journal of Physical Chemistry C 124, 28448–28457. <https://doi.org/10.1021/acs.jpcc.0c06127>
